# Evaluation frameworks for digital nursing technologies: analysis, assessment, and guidance. An overview of the literature

**DOI:** 10.1186/s12912-021-00654-8

**Published:** 2021-08-17

**Authors:** Tobias Krick

**Affiliations:** 1 University of Bremen, SOCIUM Research Center on Inequality and Social Policy, Mary-Somerville-Straße 3, 28359 Bremen, Germany; 2 University of Bremen, High-profile Area of Health Sciences, Bremen, Germany

**Keywords:** Framework, Evaluation, Digital, Technology, Care, Nursing

## Abstract

**Background:**

The evaluation of digital nursing technologies (DNT) plays a major role in gaining knowledge about certain aspects of a technology such as acceptance, effectiveness, or efficiency. Evaluation frameworks can help to classify the success or failure of a DNT or to further develop the technology. In general, there are many different evaluation frameworks in the literature that provide overviews of a wide variety of aspects, which makes this a highly diverse field and raises the question how to select a suitable framework. The aim of this article is to provide orientation in the field of comprehensive evaluation frameworks that can be applied to the field of DNT and to conduct a detailed analysis and assessment of these frameworks to guide field researchers.

**Methods:**

This overview was conducted using a three-component search process to identify relevant frameworks. These components were (1) a systematized literature search in PubMed; (2) a narrative review and (3) expert consultations. Data relating to the frameworks’ evaluation areas, purpose, perspectives, and success definitions were extracted. Quality criteria were developed in an expert workshop and a strength and weakness assessment was carried out.

**Results:**

Eighteen relevant comprehensive evaluation frameworks for DNT were identified. Nine overarching evaluation areas, seven categories of purposes, five evaluation perspectives and three categories of success definitions could be identified. Eleven quality criteria for the strengths and weaknesses of DNT-related evaluation frameworks were developed and the included frameworks were assessed against them.

**Conclusion:**

Evaluators can use the concise information and quality criteria of this article as a starting point to select and apply appropriate DNT evaluation frameworks for their research projects or to assess the quality of an evaluation framework for DNT, as well as a basis for exploring the questions raised in this article. Future research could address gaps and weaknesses in existing evaluation frameworks, which could improve the quality of future DNT evaluations.

**Supplementary Information:**

The online version contains supplementary material available at 10.1186/s12912-021-00654-8.

## Background

A large number of digital nursing technologies (DNTs) are currently being developed and tested in nursing practice [1, 2]. These technologies offer promising opportunities to address existing societal challenges such as the shortage of skilled workers or the increasing demand for long-term care [3].

This article refers to digital nursing technology (DNT) as defined by Krick et al. 2019 [2] and Krick et al. 2020 [4]. DNT are technologies that fulfil one or all of the following criteria: i) “support the immediate action of a caregiver”; or ii) “contribute to the self-reliance of the person in need of care in such a way that direct on-site care assistance can be avoided”; or iii) “substitute the nursing support by using technology”, or iv) “support the training or education of nurses” [4]. The focal points of this article are the aspects i-iii. DNT’s can, for example, be information and communication technologies, robots, sensors, monitoring technologies, assistive devices, ambient assisted living technologies, virtual reality or tracking technologies [1, 2].

Professional nurses point to the need for improved technological support in direct care to reduce physical strain and psychological stress [5], which e.g. could have a long-term impact on retention in the profession. On the other hand, use and acceptance in actual clinical support appears to be rather low [6, 7].

The reasons for the lack of acceptance and usage can be very diverse, since DNT are complex interventions [8]. Specific reasons for non-adoption can be that technologies are not user-friendly (low usability) [5] or have no obvious perceptible benefit for actual work practice (job relevance, perceived usefulness) [5]. Privacy issues or cost concerns may also bea major concern for persons in need of care [9]. Scientific evaluations that provide information on technologies from different perspectives and viewpoints could help us to understand the bigger picture of DNT success and provide important insights on specific impact factors. Evaluation results can, for example, help decision-makers to facilitate the process of system implementation [10]. Evaluation conducted during the development process also has the potential to prevent system failures and misdevelopments [10].

Comprehensive evaluation frameworks that clearly present important aspects of evaluation play a significant role in supporting researchers, decisionmakers and developers in this process. Evaluation frameworks can be used to provide a structure for the evaluation of DNT as they provide information and definitions of technology success, evaluation areas, methods, and tools. In this way, they “facilitate a systematic approach” [11] in DNT evaluation. The information provided by evaluation frameworks can enable different stakeholders to gain a common understanding of the evaluation process and help identify and decide on appropriate objectives and methods. This can help mediate the fit between research and practice-oriented approaches to evaluation [11].

This article focusses on comprehensive evaluation frameworks that include information on multiple evaluation areas. There is a wide variety of frameworks in the literature and most of them are highly heterogeneous in content, approaches, and methodologies. Identifying and deciding on the appropriate evaluation framework can be very challenging, as there is no suitable overview of evaluation frameworks in the field of DNT available in the literature. It is also difficult to see how these frameworks differ in terms of purpose, and areas of evaluation, and their definition of success. Orientation must be provided on these possibilities, and this overview was conducted to this end.

## Objective and research question

The objectives of this article are to provide orientation on existing comprehensive evaluation frameworks that can be applied in the field of DNT and to conduct a detailed analysis and assessment of these frameworks. The aim is to contribute to the discussion and understanding of what constitutes a good (DNT) evaluation framework and to offer field researchers guidance in the selection and application of evaluation frameworks.

This article is thus guided by the following research questions: (i) Which comprehensive evaluation frameworks that can be applied to DNT evaluation are available in the literature? (ii) What purposes, perspectives, and definitions of success are described in these frameworks? (iii) What are the strengths and weaknesses of the included frameworks? (iv) Which areas of evaluation are represented in the frameworks and where are the most overlaps and differences between these frameworks?

## Methods

### Search process

**Fig. 1:**
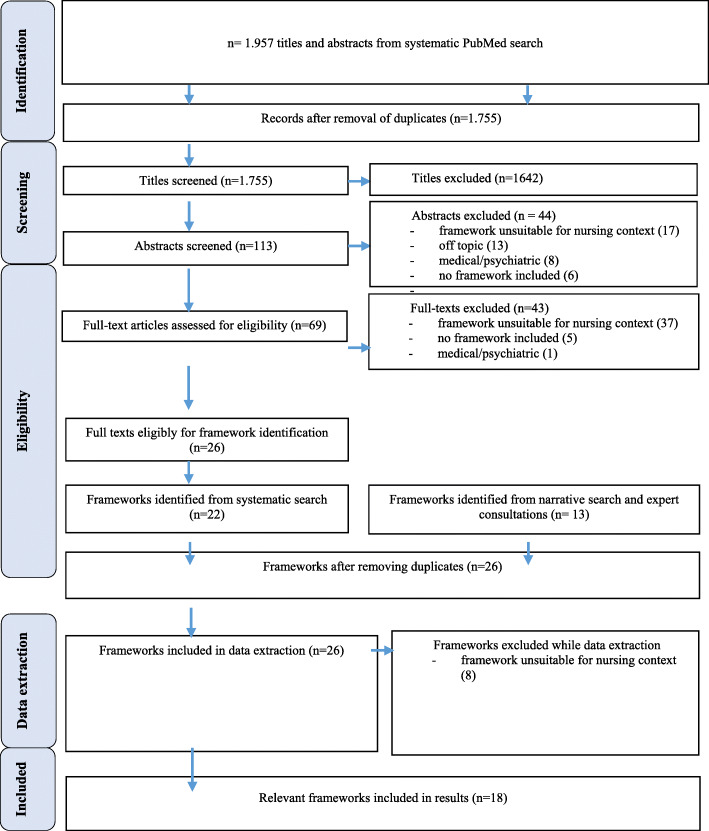
Search results and framework selection process

This overview [12] was conducted using three different search components (roman numerals). The goal was to identify frameworks of relevance to the nursing context according to the inclusion and exclusion criteria described further below. The search components comprised: (i) a systematized literature search conducted in PubMed; (ii) a narrative review was carried out by searching google scholar, screening already identified literature [4] and reference lists of the systematically identified articles; (iii) information on relevant frameworks collected from four experts in the field of evaluation from the German “Cluster Zukunft der Pflege” (Future of Care Cluster) [13]. In this joint project, regular evaluations of digital nursing technologies have been and are carried out over the period 2017–2022. The search terms of the systematized literature search can be found in Table 1. The complete search strategy, including all three components, is shown in Fig. 1.

**Table 1:** Search terms of the systematic search in PubMed

Term		Term		Term	Hits
Framework	AND	Evaluation	AND	Technol*	1381
Framework		Evaluation		ICT	24
Framework		Evaluation		Robot	48
Framework		Evaluation		Sensor	135
Framework		Evaluation		Telecare	10
Framework		Evaluation		Telemed	97
Framework		Evaluation		Digital	260
					1955

### Eligibility criteria for systematic search

Scientific papers included in the search had to have been published between 2005 and 2020 in English. A summary and full text had to be available. All information was gathered in March 2020, which limits the inclusion period from January 2005 to March 2020.

The studies in question had to (i) either apply an evaluation framework to a (nursing) technology or describe the development of an evaluation framework. The selected frameworks had to (ii) be at least based on either a literature study, an empirical evaluation, or an expert survey. They had to (iii) be directly related to technologies in (nursing) care, or comparable fields like the evaluation of digital health applications in general, but they had to be potentially suitable for the evaluation of DNT. (iv) The technology specific focus must lie on Information and Communication Technologies (ICT), telemedicine, telecare, sensor technology or robotics (or their sub-sectors), as these are the most common technology areas in nursing care (definitions by Krick et al. 2019) [2]. (v) The frameworks could refer to the evaluation of different stages of the life cycle of a technology [14] e.g. before, during or after implementation.

Exclusion criteria were: articles (i) focussing on frameworks for the evaluation of specific medical technology fields (e.g. radiology, surgery) with no relation to nursing; (ii) from developing countries or underdeveloped health systems; (iii) that described study protocols; (iv) with overviews that only present categorical systems without creating a framework; (v) focussing on frameworks related to technologies for education or training; (vi) focussing on fitness applications, wellness applications or applications for general disease prevention; (vii) in a psychiatric context; (viii) that are not comprehensive, i.e. only focus on individual areas such as economy or acceptance or satisfaction or usability; (ix) which refer exclusively to the implementation and not the evaluation of the implementation.

### Identifying relevant frameworks

All systematic search results were imported into EndNote X8 and reimported into the Excel screening workbook by VonVille [15]. A multi-step screening process was performed. The first step included screening 100 titles and 100 abstracts. The eligibility criteria were then refined. All titles were screened in the second step and the remaining abstracts in the third step. The eligibility criteria were then refined again before screening the full texts. If an identified article only applied a framework, for which the development is described in another article, this was an intermediate step for the identification of the framework. The original article describing the framework was than identified and included in the further steps of the analysis.

The narrative search was performed with the knowledge and eligibility criteria of the first screening process, which enabled a much more precise identification process. Google scholar was searched with the terms “framework” AND “evaluation” AND “nursing”. Articles were screened and reference lists were also examined, snowballing through the reference lists of these articles. Reference lists of the systematically identified studies and literature from a previous search [4] were also included. This method of snowballing is important for such complex search fields. It helped to obtain all relevant information on frameworks as a supplement to those not found in the systematic approach [16].

The expert consultation additionally focussed on the identification of relevant frameworks. The experts were invited to name frameworks known to them and list all frameworks used in their projects to evaluate digital nursing technologies. The whole search and identification process can be found in Fig. 1.

### Data extraction

#### Purpose, perspective, and success definitions

The first step for data extraction was to screen all full texts of the frameworks for the technology group to which they refer, the stated purpose and the evaluation perspective [17], as well as the success definition/description (ii). This article defines the purpose of a framework as the description of what the framework is intended to achieve. The perspective describes the viewpoint from which the framework was developed and thus the viewpoint from which the evaluation results could be interpreted. The analysis of “success” focused on the definitions or descriptions of what the articles mean by “success” or “successful technology”. A qualitative synthesis was conducted to identify and categorize the included purposes, perspectives and success definitions by using textual narrative synthesis [18].

#### Strengths and weaknesses

A strengths and weaknesses analysis of the frameworks under consideration was also carried out to answer research question (iii). The criteria for evaluating the frameworks were developed in an expert workshop with experienced researcher in the field of evaluation. The criteria are listed in Table 2. This approach was chosen because there are no universal quality criteria for the strengths and weaknesses of DNT related evaluation frameworks in the literature.

**Table 2:** Guiding categories for assessing the strengths and weaknesses of the frameworks

Guiding Category	Content
Focus of the framework	Description of the specific focus of the framework. This can include a - description of the purpose (and the addressed question) - the application setting - the technology (area)
Illustration	- Clarity/ complexity of illustration - Visualization of connections and relationships within the framework
Terminology	- Transparent definitions of terms and key concepts
Instructions for use	- Concrete application strategy and instructions for use - Instruction on how the results can be interpreted
Scientific quality	- Transparency of development process - Reflection of the limitations of the framework - Transferability of the framework (Settings, technologies, questions)

#### Areas of evaluation

To answer research question (iv) all frameworks were screened for similarities in their categorization systems. Most frameworks used different sorting systems and systematization logics. An iteratively developed data extraction form was drawn up in Excel and piloted with three frameworks. It was decided to assign all evaluation topics of the frameworks to the generic categories “Top Category” “Subcategories” and “Specification” (Additional file 1). This step was necessary, because there is no universal systematization to categorize the content of evaluation frameworks, but there is a kind of hierarchy that can be found in these frameworks.

#### Charting the data

All extracted information on the technology group to which the frameworks refer, the stated purpose and the evaluation perspective, as well as the definition/description of success (research question ii), were charted in excel and listed with the respective framework.

To answer research question (iv) all identified top categories were analyzed to build overarching top categories that were used for the systematization of these frameworks. The analysis of all top categories of the 18 frameworks resulted in 9 generic top categories, which were then defined and formed the basis for further analysis. The definitions of these categories were generated inductively and iterative while analyzing all included evaluation aspects of the frameworks (analysis in Additional file 2; definitions in Table 3). Despite this process a non-overlapping categorization of these categories was not possible due to the complexity of the frameworks content and the interconnectedness of different categories.

**Table 3:** Definitions of the DNT evaluation areas

**Focus**	**Product/Technology**	**Objective Value/Effect**	**Individual**	**Organization**
This area includes what the technology focuses on in terms of its objectives and purpose and the problems and needs it aims to solve for a specific target group in a specific setting.	This area includes all aspects of the technology itself. This ranges from visual appearance to functionality and certain specific technological aspects such as interoperability. (However, there is also an interface to the category “individual”, because certain individually perceived aspects are covered here, such as usability and access).	This category includes the relevant information on evidence, aspired values as well as intended and unintended effects of the technology.	This area includes reactions and perceived impressions, as well as the behaviour and the relationship of individuals towards the technology.	This area includes aspects that are relevant in the relationship between the technology and an organization.
**Societal**	**Ethics**	**Economics**	**Strategic**	
This area includes relevant aspects of the technology in a societal context (e.g. political, juridical, regulatory, or socio-cultural aspects - Overlaps with the area of ethics are possible).	This area includes relevant ethical standards and ethical implications to be considered in relation to the technology.	This area includes relevant economic aspects for the technology (e.g. business model, price, economic evaluation).	This area includes strategic aspects that may be relevant for the introduction and dissemination of the technology.	

In the next step, the extracted content of the subcategories from the frameworks (Additional file 2) was analyzed to identify similarities and differences related to the newly built top categories (Table 3). During the extraction of the category “specifications”, these were found to be vastly diverse and, hence, not suitable for any standardization. Therefore, only the content of the subcategories was included in the mapping process. Where there were no subcategories, the top categories were included into this step. This was the case for the: Design and Evaluation of DHI Framework [14], Evaluation Framework for Fit-For-Purpose Connected Sensor Technologies [19], Digi-HTA [20], CISSM [21] (in parts because there were only specifications in the form of specific questions for the top categories).

If the subcategories corresponded to a completely different sorting logic as the other frameworks and therefore did not contain any evaluable information, the “specifications” were evaluated if they contained valid information. This was done for the: Khoja–Durrani–Scott Evaluation Framework [22], the layered telemedicine implementation model [23]; and the Comprehensive evaluation framework for telemedicine implementation [24].

All charting results can be found in (Additional file 2). The assignment may differ from their logic in the representation to the original assignment because the frameworks used different sorting systems and logics, which were unified in this representation. Models and frameworks often develop their own categorization logic. There is no uniformly recognized logic.

## Results

### Search results

The systematic search in PubMed generated 1957 hits. After removing the duplicates, 1.755 remained for screening the titles. The abstracts of 113 articles were chosen for screening, yielding 69 full-texts eligible for full-text screening. The systematic search generated a total of 22 articles to be analyzed for relevant frameworks. The narrative search and the expert consultations yielded 13 articles with frameworks. There remained 26 frameworks for the final analysis process, after sorting out the duplicates generated by the different search processes. Eight further frameworks were discarded during the data extraction process because detailed analysis revealed that they did not meet the eligibility criteria. This left 18 frameworks filtered out for the final analysis (Fig. 1).

### Analysis results

Eighteen comprehensive evaluation frameworks that can be applied to DNT are presented in the results section. According to the technology categories there are *n* = 7 frameworks related to information and communication technologies (with different sub-sectors), *n* = 3 frameworks for telemedicine/telecare, and *n* = 1 framework for sensor technologies. The remaining frameworks were generalistic frameworks from the areas of digital health (n = 3), health (and care) technologies (*n* = 2), e-health (n = 1) and clinical informatics (n = 1). The classification was based on the technologies derived from the articles with reference to the definitions of technology categories from Krick et al. 2019 [2] (Table 3). The final selection of frameworks can be found in Table 4.

**Table 4:** Included Frameworks and technology categories

**Information and Communication technologies**	Infoway benefits evaluation Framework [25]
	Health Information Technology Evaluation Framework (HITREF) [26]
	Hospital Information System Success Framework [27]
	Development of an Evaluation Framework for Health Information Systems (DIPSA Framework) [28]
	Human, Organization, Process and Technology-fit (HOPT-FIT) [29]
	Clinical Information Systems Success Model (CISSM) [21]
	Adapted nursing care performance framework [30]
**Telemedicine/Telecare**	Model for Assessment of Telemedicine (MAST Manual) [31]
	Comprehensive evaluation framework for telemedicine implementation [24]
	The layered telemedicine implementation model [23]
**Sensor Technologies**	Evaluation Framework for Fit-For-Purpose Connected Sensor Technologies [19]
**Digital Health**	Design and Evaluation of DHI Framework [14]
	Health technology assessment framework for digital healthcare services (Digi HTA) [20]
	Digital Health Score Card [32]
**Health (and care) technologies**	Health Technology Adoption Framework [33]
	Nonadoption, Abandonment, Scale-up, Spread, and Sustainability Framework (NASSS Framework) [34]
**E-health programs**	Khoja–Durrani–Scott Framework for e-Health Evaluation [22]
**Clinical informatic interventions**	RE-AIM (Reach, Effectiveness, Adoption, Implementation, and Maintenance) (expanded to clinical informatics)) [35]

### Purpose and perspectives of the frameworks

The purposes and the perspectives of the selected frameworks were analyzed to answer research question (ii). Although these frameworks have the common purpose of assessing digital technologies using specific assessment categories, the purposes described beyond this differ. The detailed assessment can be found in Table 5. The qualitative synthesis identified 7 overarching categories of purposes:

1. Help and guide researchers (design and evaluation process) [14, 22, 25–27, 32–35]
2. Identify success and failure factors (and help to manage them) [14, 19, 21, 23, 27, 34]
3. Assess the performance/success of a technology (outcomes, impact, errors, deficiencies) [28–31]
4. Make the results comparable [19]
5. Contribute to the quality and development of the technology [31, 34, 35]
6. Support the implementation of a technology [33–35]
7. Help in decision-making [20, 24, 31]

**Table 5:** Analysis and assessment of the frameworks

	Analysis				
Technology Field	Framework	Authors/Year	Perspective	Stated Purpose	Success Definition/Description
**Information and Communication technologies** (Health Information Systems (HIS))	Infoway benefits evaluation Framework	Francis Lau et al. 2007 [25]	**Investment programs** for digital technologies (to guide evaluations)	1. Provide a high-level evidence based model to guide subsequent field evaluation	Success measured by analysing the results of the evaluation (Factors based on the van der Meijden et al. model [36])
**Information and Communication technologies** (Health Information Technologies (EHR))	Health Information Technology Evaluation Framework (HITREF)	Sockolow et al. 2012 [26]	**Universal perspective** (mainly influenced by health services research and informatics)	1. Conceptual tool for framing evaluations studies in assessing EHR-based implementations in organizational, systematic, and environmental contexts 2. Displaying evaluation criteria	No success definition (measuring the success by analysing the results of the evaluation)
**Information and Communication technologies** (Hospital Information Systems)	Hospital Information System HIS Success Framework	Sadoughi et al. 2013 [27]	**Universal perspective**	1.Identification of Hospital Information System success and failure factors and the evaluation methods of these factors	Success as a dynamic concept. Success is when the technology achieves its intended purpose. (+ time, budget, and user satisfaction)
**Information and Communication technologies** (Integrated Health Information Systems (IHIS))	Development of an Evaluation Framework for Health Information Systems (DIPSA Framework)	Stylianides et al. 2018 [28]	**Healthcare Organization**	1.Evaluation framework for hospitals utilizing IHIS to help identify any existing deficiencies in the system	No success definition (measuring the success by analysing the results of the evaluation)
**Information and Communication technologies** (Health Information Systems)	Human, Organization, Process and Technology-fit (HOPT-FIT)	Yusof 2019 [29]	**Healthcare Organization** (focus on technology induced errors)	1. Evaluate HIS performance and efficiency 2. Systematically guide error evaluation 3. Describing the Human-Organization-Process-Technology fit	No success definition (measuring success with the included dimensions of HIS success)
**Information and Communication technologies** (Clinical Information Systems (CIS))	Clinical Information Systems Success Model (CISSM)	Garcia-Smith & Effken 2013 [21]	**Nurse’s perspective**	1. Framework for evaluating CIS success from the nurse’s perspective	Success = net benefit (“degree to which a nurse believes that using a particular system enhances job performance”)
**Information and Communication technologies** (Information and Communication technologies for nurses)	Adapted nursing care performance framework	Rouleau et al. 2017 [30]	**Nurse’s perspective**	1. Illustrate how ICTs interventions influence nursing care and impact health outcomes	No success definition (measuring the success by analysing the results of the evaluation)
**Telemedicine/Telecare**	Model for Assessment of Telemedicine (MAST Manual)	Kidhom et al. 2010 [31]	**Universal perspective** (user-based decision making, research)	1. Describe effectiveness 2. Contribution to quality of care of telemedicine applications 3. Produce a basis for decision making	No success definition (measuring the success by analysing the results of the evaluation)
**Telemedicine/Telecare**	Comprehensive evaluation framework for telemedicine implementation	Chang 2015 [24]	**Universal perspective** (decision making for individuals, organizations, and communities)	1. Summarising important themes for the evaluation of telemedicine systems 2. Support related stakeholders’ decision-making by promoting general understanding, and resolving arguments and controversies	Long-term implementation
**Telemedicine/Telecare**	The layered telemedicine implementation model	Broens et al. 2007 [23]	**Universal perspective** (the focus on individual determinants/ perspectives changes throughout the development life cycle)	1. Detailed classification of the determinants of the success of future telemedicine implementations	Successful implementation (“putting an idea or a concept into actual practice”)
**Sensor Technologies** (Connected Sensor Technologies: including wearables. biosensors)	Evaluation Framework for Fit-For-Purpose Connected Sensor Technologies	Coravos et al. 2020 [19]	**Healthcare System Perspective** (users and other stakeholders)	1. Working evaluation framework that reflects different types of risks 2. Framework is conducted to better manage these risks 3. Make information on sensor technologies more comparable and understandable	No success definition (success could be measured by analysing the results of the evaluation and comparing them with the standards for connected sensors)
**Digital Health** (Digital Health Interventions (DHI))	Design and Evaluationof DHI Framework	Kowatsch et al. 2019 [14]	**Universal perspective** (researchers and practitioners)	1. Framework for the design and evaluation of DHI 2. Showing evaluation criteria and implementation barriers to be considered during the life cycle phases of DHI 3. Support researchers and practitioners from conception to large-scale implementations	A successful DHI needs to consider “the selection of suitable evaluation criteria and the overcoming of implementation barriers”
**Digital Health** (Digital Healthcare Services: mHealth, AI, and robotics)	Health technology assessment framework for digital healthcare services (Digi HTA)	Jari et al. 2019 [20]	**Healthcare System Perspective** (decision making)	1. Inform decisionmakers in order to better support the introduction of new health technologies	No success definition (success could be measured by analysing the results of the evaluation)
**Digital Health** (Digital Health Technologies)	Digital Health Score Card	Mathews et al. 2019 [32]	**Universal perspective** (multi-stakeholder approach)	1. Multi-stakeholder approach to objectively evaluate digital health solutions	Measuring the success by analysing the results of the evaluation (Success as the successful delivery of validated digital health solutions)
**Health (and care) technologies**	Health Technology Adoption Framework	Poulin et al. 2013 [33]	**Healthcare Organization**	1. Framework with clear, user-validated criteria for evaluating new health technologies for adoption at the local level	No success definition (success could be measured by analysing the results of the evaluation)
**Health (and care) technologies**	Nonadoption, Abandonment, Scale-up, Spread, and Sustainability Framework (NASSS Framework) [34]	Greenhalgh et al. 2017	**Universal perspective**	1. Framework to help predict and evaluate the success of a technology-supported health or social care program 2. Help to design, develop, implement, scale up, spread, and sustain technology-supported health or social care programs by identifying key challenges in different domains and the interactions between them	Adoption, scale-up, spread, and sustainability of a technology
**E-health programs**	Khoja–Durrani–Scott Framework for e-Health Evaluation	Khoja et al. 2013 [22]	**Universal perspective** (included tools usable for managers, healthcare providers, and clients)	1. Comprehensive Framework to show relevant themes for e-health evaluation	No success definition (measuring the success by analysing the results of the evaluation)
**Clinical informatic interventions**	RE-AIM (Reach, Effectiveness, Adoption, Implementation, and Maintenance) (expanded to clinical informatics)	Bakken & Ruland 2009 [35]	**Healthcare Organization** (implementation in organizational practice)	1. Used to design, implementation, evaluation, and reporting of clinical informatics with a goal of translation of research into practice	No success definition (measuring the success by analysing the results of the evaluation)

A further classification of the purposes could be made by dividing them into two main categories. (A) knowledge-oriented purposes (1–4) that mainly indicate that the frameworks and their use serve to generate a certain form of knowledge. (B) practice-related purposes (5–7). Theses purposes could be summarized as application-oriented knowledge as they indicate that the knowledge will be used for a specific action such as development, implementation or decision making.

For a deeper understanding of the purpose of a framework, it is necessary to also analyze the perspective that the framework takes as it might have an influence on how the framework is intended to achieve that purpose.

Of the 18 frameworks, *n* = 9 had a universal perspective, which means that they can be applied to different perspectives or remain neutral thus leaving the decision to the evaluator. The remaining nine frameworks indicate or define for themselves, that they were developed from a specific viewpoint, and that the results could be interpreted from this viewpoint. Of these nine, *n* = 4 take the perspective of a healthcare organization, *n* = 2 describe the healthcare system as a perspective, n = 2 have the nurse’s perspective as a viewpoint and *n* = 1 is developed from an investment program perspective.

### Success definitions/descriptions

The frameworks were also scrutinized for definitions or descriptions of what is meant by “success” or “successful technology” in order to gain a better understanding of the differences that might exist (Table 5). Most of the frameworks do not have an explicit definition of success [19, 20, 22, 26, 28–33]. In such cases, the evaluation criteria described in the frameworks and the resulting interpretation of the evaluation results could be used to make a statement about whether or not the technology in question was successful.

The qualitative synthesis of the success definitions/ descriptions of success identified three categories:

1. Success is when the technology achieves its intended purpose [27]
2. Success means achieving implementation, dissemination and/or sustainability of a technology [14, 23, 24, 34]
3. A successful technology must generate a net benefit [21]

### Strengths and weaknesses of the frameworks

The strengths and weaknesses of the frameworks under consideration were assessed in order to contribute to a better understanding of what constitutes a good (DNT) evaluation framework and answer research question (iii). Previously developed quality criteria for DNT Frameworks were used for the assessment (as described in the methods section). A detailed analysis of the assessment can be found in Table 6. (+) stands for strength and (−) denotes a weakness in a certain assessment area.

**Table 6:** Strengths and Weaknesses

			+ = strength			- = weakness			+/− = strength and weakness					
			Criteria									Summary		
**Frameworks**	Description of the purpose (and the addressed question(s)	Description of the application setting	Description of the technology (area)	Clarity/ complexity of illustration	Visualization of connections and relationships within the framework	Transparent definitions of terms and key concepts	Concrete application strategy and instructions for use	Instruction on how the results can be interpreted	Transparency of development process	Reflection of the limitations of the framework	Transferability of the framework (Settings, technologies, questions)	+	–	+/−
Health Technology Adoption Framework [33]	+	+	+	+	–	+	+	+	+	+	+/−	9	1	1
Clinical Information Systems Success Model (CISSM) [21]	+	+	+	+	+	+	+	+	+	–	+/−	9	1	1
Nonadoption, Abandonment, Scale-up, Spread, and Sustainability Framework (NASSS Framework) [34]	+	+/−	+/−	+	+	+	+/−	+	+	+	+	8	0	3
Health Information Technology Evaluation Framework (HITREF) [26]	+	+/−	+	+	+	+	+	–	+	–	+	8	2	1
Evaluation Framework for Fit-For-Purpose Connected Sensor Technologies [19]	+	+/−	+	+	–	+	+	+	+	–	+	8	2	1
Hospital Information System Success Framework [27]	+	+	+	–	–	+	+	–	+	+	+	8	3	0
Adapted nursing care performance framework [30]	+	+/−	+	+	+	+	–	+/−	–	+	+	7	2	2
The layered telemedicine implementation model [23]	+	+/−	+	+	+	+	+/−	–	+	+	–	7	2	2
Model for Assessment of Telemedicine (MAST Manual) [31]	+	+/−	+	–	–	+	+	–	+	+	+	7	3	1
Health technology assessment framework for digital healthcare services (Digi HTA) [20]	+	+/−	+	–	–	+	+	+	+	–	+	7	3	1
Infoway benefits evaluation Framework [25]	+	+/−	+/−	+	+	+	–	–	+	–	+	6	3	2
Design and Evaluation of DHI Framework [14]	+	+/−	+/−	–	–	+	+	–	+	+	+	6	3	2
RE-AIM (Reach, Effectiveness, Adoption, Implementation, and Maintenance) (expanded to clinical informatics)) [35]	+	+/−	+/−	–	–	+	+	+	+	–	+	6	3	2
Development of an Evaluation Framework for Health Information Systems (DIPSA Framework) [28]	+	+	+	–	–	+	–	–	+	–	+	6	5	0
Khoja–Durrani–Scott Framework for e-Health Evaluation [22]	+	+/−	+/−	–	+	+	–	–	+	–	+	5	4	2
Digital Health Score Card [32]	+	+/−	+/−	+	+	+	–	–	–	–	–	4	5	2
Human, Organization, Process and Technology-fit (HOPT-FIT) [29]	+	+/−	+	+	+	–	–	–	–	–	–	4	6	1
Comprehensive evaluation framework for telemedicine implementation [24]	+	+/−	+	+	–	–	–	–	–	–	–	3	7	1

The assessment revealed differences and similarities between the frameworks under study. All frameworks included a description of their intended purpose and the question(s) addressed. Of the 18 frameworks, 14 do not explicitly describe an application setting, since these frameworks were developed with generic setting approaches. They therefore received a (+) in the evaluation for the universality of the setting and at the same time a (−) because they are not specifically related to a setting. However, most frameworks describe specific technology areas on which they focus. Only six have universal designs, for potentially different technologies, so that they have been assessed with a (+) for universality and a (−) for being non specific. Due to their elaboration, *n* = 12 of the frameworks are easily transferable to other contexts. *N* = 11 frameworks have a very clear visual presentation and *n* = 9 include a visual representation of connections and relationships of individual aspects within the framework. On the other hand, this means that *n* = 7 frameworks were not without visual weaknesses and n = 9 frameworks did not show visual connections between the aspects with which they were concerned. Almost all frameworks (*n* = 15) included transparent definitions of terms and key concepts and are transparent in terms of the development process (*n* = 14). However, many of the frameworks have weaknesses in applicability, clear guidance, and assistance for the interpretation of the results. Only *n* = 9 frameworks are strong in the description of an application strategy and instructions for use and only *n* = 6 include soundadvice on how to interpret the results. Furthermore, many articles on the frameworks do adequately discuss weaknesses and limitations - if at all(*n* = 11). The frameworks with the highest scores across all assessment categories were:

1. Health Technology Adoption Framework [33] strengths n = 9, weaknesses n = 1 (no visualization of connections and relationships within the framework) and strength/weaknesses n = 1 (transferability limited to surgical context).
2. CISSM [21] strengths n = 9, weaknesses n = 1 (no description of the limitations) and strength/weaknesses n = 1 (transferability limited to hospital context).
3. NASSS Framework [34] strengths *n* = 8, and strengths/weaknesses *n* = 3 (no clear focus on an application setting, no clear focus on a technology (area), and no applicable strategy using the framework.)

The detailed analysis of the assessment can be found in Table 6. More detailed descriptions of the strengths and weaknesses can be found in Additional file 3.

### Areas of evaluation in relation to the assigned perspectives

A detailed analysis of the areas of evaluation included in the frameworks was carried out to answer research question (iv). The analysis resulted in definitions for nine evaluation areas that are described in Table 3.

Table 7 shows a comparison of the frameworks regarding the evaluation areas they cover. The results of the analysis of these areas indicate where the frameworks have their main areas of focus. The colour coding in the table signals that a framework covers a certain area. The allocation was based on the definitions and the sorting logic described in the Methods section above. The specific perspective described for the frameworks was also included in the table to crosscheck whether it is possible to make generalized statements about the existence of certain evaluation areas in relation to the perspective taken.

**Table 7:**
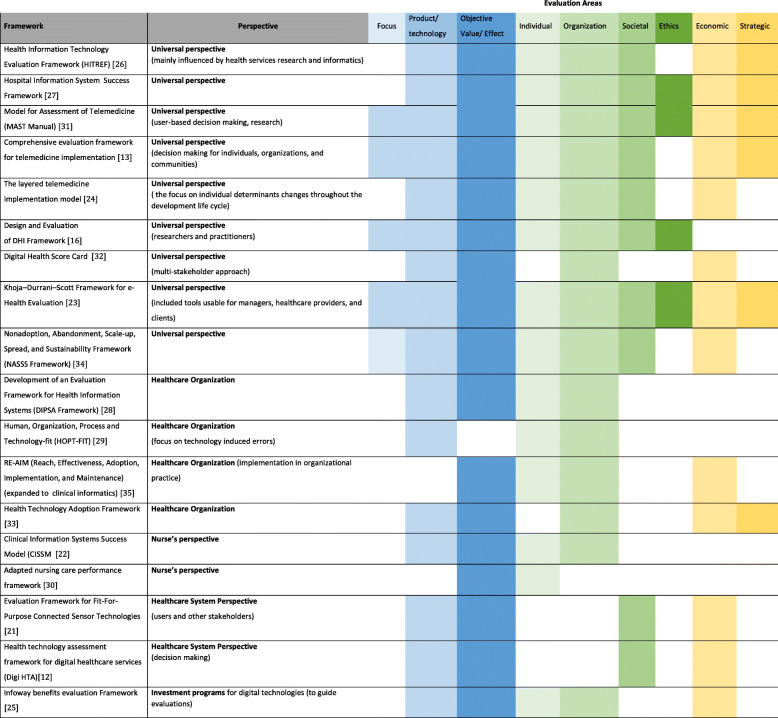
Frameworks with evaluation areas and perspectives

Most frameworks (*n* = 17) contained evaluation aspects of the area of objective value/effect. Also, the evaluation of the specific product/technology aspects (*n* = 16), aspects of the organization (*n* = 15) and the relationship of individuals to the technology (*n* = 14) was largely represented.

Societal (*n* = 10) and strategic (*n* = 6) aspects, as well as ethical aspects (*n* = 4), were not as frequent. A closer look reveals that these aspects are particularly rare when the perspective described is the healthcare organization or the nurses. At the same time, these aspects are jointly represented three times in the assessment if the perspective “universal” was assigned (in the HIS Success Framework [27], MAST Manual [31] and the Khoja–Durrani–Scott Framework for e-Health Evaluation [22]).

Overall the evaluation area “focus” is not as frequently represented. Only *n* = 5 frameworks contain aspects of this areas. All frameworks containing this aspect come from the “universal” perspective. The area covers evaluation aspects that can be used as starting point for the design of a DNT or DNT evaluation by conducting a “needs analysis” (related to the addressed problems and needs of a target group).

Frameworks for which a universal perspective has been described (*n* = 9) also cover more areas more often in general (coverage of 7,2 areas on average). While frameworks for which a healthcare organization perspective is described (*n* = 4) only cover 4 areas on average (none of them covers the areas focus, societal or ethics), and frameworks covering the nurse’s perspective (*n* = 2) only cover 2 and 4 areas respectively (none of them covers the focus, societal, ethics, economic or strategic aspects). The frameworks with a healthcare system perspective (n = 2) cover 4 areas on average (none of them covers the focus, individual, organization, ethics, or strategic areas) and the only framework with an investment program perspective covers 5 areas (does not include the areas focus, societal, ethics or strategic). A mapping of the content of the frameworks to the evaluation areas was carried out and can be found in Table 6.

## Discussion

The aim of this study was to contribute to a better understanding of what constitutes a good (DNT) evaluation framework and to guide field researchers in the selection and application of evaluation frameworks. This aim resulted in four research questions: (i) Which comprehensive evaluation frameworks that can be applied to DNT evaluation are available in the literature? (ii) What purposes, perspectives, and definitions of success are described in these frameworks? (iii) What are the strengths and weaknesses of the included frameworks? (iv) Which areas of evaluation are represented in the frameworks and where are the most overlaps and differences between these frameworks?

Eighteen different comprehensive evaluation frameworks were identified that met the inclusion criteria in the field of DNT. Unlike other overviews of evaluation frameworks, which either had a very technology-specific focus (e.g. on health information systems [37]) or a different thematic orientation (e.g. on HTA [38]), this article took a broad approach on comprehensive frameworks for DNT.

This led to the identification of technology-specific frameworks *n* = 11 (Information and Communication technologies, telemedicine/telecare, and sensor technologies) – as well as more generalistic frameworks addressing digital health (*n* = 3), health (and care) technologies (*n* = 2), e-health (n = 1) or clinical informatics (n = 1). The identified frameworks and their main features are listed in the Tables 4–7. These may serve as a good overview and starting point for researchers to select an appropriate framework.

It should be noted, however, that although a specific definition of DNT was used, there is inevitably an overlap to other different themes like e-health or digital health which makes a general distinction very difficult. The frameworks for specific technology categories in this article only cover a part of the technologies that can be subsumed under DNT. For example, evaluation frameworks for monitoring technologies, assistive devices or ambient assisted living, are not included [2]. This is so as to avoid too much heterogeneity of technologies in this article and the technologies included make up a significant proportion of the DNTs discussed in the literature [2]. Also, the generic frameworks can potentially be used for the evaluation of further technologies.

To distinguish, differentiate and select relevant frameworks, researchers should look at specific assessment categories and the frameworks content. Some important aspects regarding the frameworks are covered in this article. The perspective of a framework is essential to put the definitions of success in a framework into context and to understand a potential interpretation of the evaluation categories. There should also be clarity about the purpose of the framework to apply it appropriately, and transparency about where frameworks have their respective strengths and weaknesses. All these issues were analyzed and are discussed in the following.

### Purposes

It is important to understand that although the common purpose of the frameworks dealt with here is to assess digital technologies using specific evaluation categories, the purposes described beyond this differ. Most frameworks included here were developed (i) to help and guide researchers in the design and realisation of an evaluation [14, 22, 25–27, 32–35], and/or (ii) to support the identification of success and failure factors (and to help manage them) [14, 19, 21, 23, 27, 34]. (iii) Four frameworks were specifically designed to assess the performance/success of a technology (outcomes, impact, errors, deficiencies) [28–31], but only one framework (iv) was drawn up to help to make the results comparable [19]. This could be since the other articles simply assume the comparability issue to be implicitly logical and therefore do not name it explicitly as a purpose. These four purpose categories (with the exception of the management of success and failure factors) could be summarized as knowledge-oriented purposes, indicating that the frameworks mainly serve to generate a certain form of knowledge.

Some frameworks indicate a more practice-related purpose such as (v) contributing to the quality and development of the technology [31, 34, 35], (vi) supporting the implementation of a technology [33–35] or (vii) providing help in decision-making [20, 24, 31]. These purposes can be summarized as application-oriented knowledge generation. As the classification shows, a DNT framework can have several purposes in both categories: knowledge generation and application orientation. The knowledge-oriented categories i, ii, iii, iv and the practice-related category vi are in line with seven out of eight general attributes of evaluation frameworks in healthcare identified by Bradford et al. (2019) These are 1. simplify a complex (evaluation) process, 2. provide structure (for an evaluation), 3. facilitate the evaluation process, 4. promote meaningful evaluation, 5. identify and explain outcomes, 6. generate transferable lessons, 7. identify mechanisms driving or inhibiting change [39]. Bradford and colleagues also consider it an important element that frameworks help to identify relevant stakeholders [39]. This element was not mentioned as a purpose by any of the frameworks in the present study. In general, DNT evaluation and DNT relevant evaluation frameworks can consider knowledge-oriented and application-oriented purposes. It does not make a framework better or worse if it includes only one of the categories, but having a clearly defined purpose is a quality criterion when choosing a reliable framework. Researchers must be aware of the intended purpose of a framework when choosing their evaluation approach.

### Perspective and evaluation areas

A closer look at the perspectives of the frameworks discussed here raises the question whether the perspective under which a framework has been developed, may have an impact on the evaluation categories included. The analysis of the frameworks revealed five perspectives: universal, healthcare system, healthcare organization, nurses, and investment program perspective.

Of the 18 frameworks, *n* = 9 had a universal perspective, which means that they can be applied to different perspectives, leavingthe decision is left to the evaluator. Frameworks for which a universal perspective has been described cover more evaluation areas in general (coverage of 7,2 areas on average), what supports this assumption. Frameworks for which a healthcare organization perspective is described only cover 4 areas on average (none of them covers the areas focus, societal or ethics), and frameworks with the nurse’s perspective only cover 3 areas on average (none of them covers the aspects focus, societal, ethics, economic or strategic). Those frameworks with a healthcare system perspective (*n* = 2) cover 4 areas on average (with none of them covering the areas focus, individual, organization, ethics, or strategic) and the only framework with an investment program perspective covers 5 areas (does not include the areas focus, societal, ethics or strategic). The perspective with which a framework has been developed is therefore always an important feature to consider when selecting a framework, as this could influence the evaluation aspects included.

In addition, there are several stakeholder perspectives that were not taken as the main perspective in the frameworks. These perspectives are the payors perspective, the perspective of the patient / person in need of care and the perspective of the informal caregiver. All these perspectives are particularly important in the context of DNTs. However, it should be mentioned that these perspectives are often included in the universal frameworks like the patient perspective in the MAST [31] or the 3rd party payment aspect in the comprehensive evaluation framework for telemedicine implementation [24]. In summary, when selecting and using a comprehensive evaluation framework to evaluate a DNT, the perspective of the chosen framework and the intended perspective of the evaluation should always be reflected and contrasted, as there may be a relationship between the perspective and the evaluation areas included in the framework. The frameworks with a narrow perspective, such as the nurses’ perspective, cover fewer evaluation areas overall in this study, while those with a universal perspective cover significantly more. Three examples explaining this in more detail can be found in the discussion section entitled “Discussion of the three most relevant frameworks”. If researchers require a comprehensive framework with as many evaluation areas as possible, they must choose a framework with a universal evaluation perspective.

### Success definitions/descriptions

It is not easy to define a successful digital nursing technology. Nguyen et al. (2014) argue that the success of a technology may be “disputed depending on the interests of the evaluating party.” [40] This is in line with the success description of Lau (2009) who sees it in the context of an “ongoing negotiation and adaptation of interrelationships” of the healthcare professionals involved [41]. A large multi stakeholder Delphi study conducted by McNair and colleagues (2006) [42] concluded that “success cannot be characterized along one single axis” and therefore defined success as the fulfilment or non-fulfilment of five consensus based aspects, namely (1) the wide usage in daily practice, (2) the fulfilment of the role and tasks it was planned for (in a specific environment), (3) the support of good medical practice (benefitting the patient), (4) the benefits to the healthcare organization and working conditions, (5) easy upgradability to adapt to the developments in practice [42].

Aspects 1–4 from McNair and colleagues were also identified as success definitions in the analyzed frameworks in this article (1. Success is when the technology achieves its intended purpose [27]; 2. Success means achieving implementation, dissemination and/or sustainability of a technology [14, 23, 24, 34]; 3. A successful technology must generate a net benefit [21]). This suggests that these aspects could be universal definitions of success for digital nursing technologies. Aspect 5 of the McNair study could be added as equally important.

However, looking at success from a certain perspective in a specific context might lead to additional perceptions or definitions of success – which could influence the evaluation.

In view of these differences in definition, it must be concludedthat the “success” of a DNT is a relative term, made up of various aspects and depths of success definitions, the selection of which depends strongly on the evaluation perspective. This insight is decisive for the evaluation of a DNT because it should always be reflected for whom and from which perspective an evaluation is conducted and what is considered as successful.

### Strengths and weaknesses of the frameworks

The strengths and weaknesses analysis process in this article identified framework components where more guidance would be beneficial, and which are important to consider when selecting a DNT evaluation framework. The assessment included the focus of a framework, the illustration, terminology, instructions for use and scientific quality. The strengths and weaknesses criteria were created especially for DNT evaluation frameworks although there are other quality criteria that could be applied from other healthcare fields. For example, Bradford et al. (2019) [39] provide 6 quality criteria for frameworks, most of which are similar to the criteria in this article. Bradford et al. also suggest assessing whether the frameworks help to identify and include stakeholders as well as mechanisms that drive or inhibit change – which might be also helpful selecting a framework.

The results of the framework assessment presented in this article need to be seen in a wider context. The evaluation was carried out with a view to assessing of perceived strengths and weaknesses in general. Several aspects mighthave been rated differently in other contexts, e.g., assessing the transferability of a framework to only onespecific context, as with the Health Technology Adoption Framework [33] and the CISSM [21] as a strength or a weakness depends on the viewpoint of the planned evaluation. If the context is surgical the Health Technology Adoption Framework might be the perfect choice, although it is not transferable to other contexts.

By showing and applying assessment possibilities for DNT frameworks this article gives guidance for the selection of appropriate DNT evaluation frameworks. An assessment of strengths and weaknesses in combination with the other important information presented in this article is crucial.

### Discussion of the three most relevant frameworks

This section describes the three highest rated frameworks of the strengths and weakness analysis in more detail in order to give researchers insights into the analytical dimensions described in this article. The complete data for all frameworks are summarized in Tables 4–7. The description is written in an application-oriented way to support researchers. Problematic aspects in the selection of DNT frameworks are also discussed.

### Framework 1: health technology adoption framework [33]

The Health Technology Adoption Framework shows validated criteria for assessing new health technologies for adoption at local level with a hospital focus in the surgical context. It is developed generically with respect to the technology to be assessed and can therefore be applied to several DNTs.

The evaluation categories presented focus on the product/technology, its objective value/effect in the adoption process, the evaluation of organizational aspects as well as economic and strategic aspects with respect to the (potential) adoption process. Individual, societal, ethical and aspects of the area “focus” are not covered. This distribution of the aspects considered could be related to the fact that the framework was developed from the perspective of the healthcare organization and is intended to help decision makers (e.g., nurse managers) to evaluate the suitability of new technologies as well as to facilitate smooth adoption from the perspective of the organization. This means that while aspects such as security, costs and strategic fit in the organization are considered, individual aspects like usability or acceptance are not. The Health Technology Adoption Framework covers more evaluation areas (*n* = 5) than the other three frameworks in this study with a health organization perspective (see Table 7). Overall, however, all frameworks with this perspective cover fewer areas than those with a universal perspective. This limitation regarding the evaluation areas should always be considered, when deciding on a framework.

The Health Technology Adoption Framework performs very well in the framework quality assessment. The descriptions and definitions are accurate and complete. An evaluation tool with appropriate evaluation categories, an application strategy, relevant questions, and a scoring logic is provided. The development process of the framework is also presented transparently, and the corresponding limitations are named in the article. Only the transferability to other settings is limited and there is no visual presentation of the framework. It is displayed in a table and not as a graphic. Overall, the Health Technology Adoption Framework provides a very good evaluation basis with valid assessment criteria.

### Framework 2: clinical information systems success model (CISSM) [21]

The CISSM framework is designed to assess the success of clinical information systems (CIS) from the nurses’ perspective. The framework is suitable for all DNTs from the CIS category in the hospital setting. CISSM focusses only on a specific selection of evaluation areas: product/technology, objective value/ effect, individual aspects, and aspects of the healthcare organization. The societal, ethical, economic and strategic aspects are not considered.

Compared to the second framework with the nurses’ perspective from this study (Adapted nursing care performance framework) [30], the CISSM covers two more evaluation areas. The Adapted nursing care performance framework [30] only covers the areas of the objective value/ effect and individual aspects of technology. By comparison, CISSM is the more comprehensive framework - but overall, both cover only a few of the possible evaluation areas. It is certainly critical that none of the two frameworks covers ethical aspects. This should be considered when selecting and applying them, as the ethical aspects are also important in this context. Additional ethical evaluation criteria from other frameworks could be added to fill this gap.

When selecting a framework with such a narrow perspective it should be borne in mind that aspects of the “bigger picture” might not be depicted. The specific success definition of a technology in this framework focusses solely on the nurses’ net benefit. Success in this case is the “degree to which a nurse believes that using a particular system enhances job performance”. This makes the CISSM framework particularly suitable for use when the nurses’ perspective is the object of research. If a broader spectrum of evaluation aspects is to be researched, then this framework would not be the right choice.

At the same time, however, the framework scores very well in the strengths and weaknesses assessment for this specific perspective. It has a very clear description of purpose, target setting, and technology addressed. It is very well illustrated, and the connections are visualized. The key concepts and terms a are completely defined. A procedure and evaluation matrix are provided as well as examples for the interpretation of an evaluation. The development process was fully explained.

The main shortcoming of the article on this framework is that no explanation is given about its limitations. Overall, the CISSM framework is a good basis to assess the success of clinical information systems (CIS) from the nurses’ perspective and the article provides a good basis and example for the methodology.

### Framework 3: nonadoption, abandonment, scale-up, spread, and sustainability framework (NASSS framework) [34]

The Nonadoption, Abandonment, Scale-up, Spread, and Sustainability Framework (NASSS) was designed to help predict and evaluate the success of health and care technologies and to support the design, development, implementation, scale up, spread, and sustainability of technology-supported health or social care programs by identifying key challenges in different domains and the interactions between them. As the description shows, this is the most comprehensive and generic framework of the three described. The framework does not focus on any specific technology or setting which makes it suitable for any DNT. The comprehensiveness of the framework is also reflected in the included evaluation aspects. The framework considers aspects of the focus, product/technology, objective value/ effect, individual aspects, aspects of the organization, societal aspects, and economic aspects – which makes it a compendium of evaluation options for DNTs. Only the ethics and strategic aspects as defined in this review are not covered by the NASSS Framework. Thus, among all the frameworks covered in this study, the NASSS framework is one that covers the most evaluation areas.

A special feature of this framework is that it addresses the issue of complexity. It categorizes the interpretation of the evaluation domains as simple, complicated, or complex. The level of complexity of each evaluation aspect is seen as the greatest challenge to scale-up, spread, and sustainability. No specific evaluation perspective is given, which makes it a universally applicable framework. However, this universality is also accompanied by a limitation. As no specific setting, technology or perspective is given, the researcher using it must always reflect on the individual application strategy. The NASSS Framework is not a directly applicable or formulaic instrument, which is reflected by the authors of the framework themselves. Specific additional tools to solve this problem have been published recently [43].

Apart from this, the NASSS framework scores very well in the strengths and weaknesses assessment.

Looking at all three frameworks in comparison, the question arises whether a framework with a specific perspective is always preferable to a universal framework. This question can only be answered on a case-by-case basis when assessing the fit of a framework to the research situation.

### Limitations of the article

It is important to discuss the limitations of such an extensive procedure as described in this article. A three-component search process was chosen for this article., namely (1) a Systematic search in PubMed, (2) a narrative search in Google Scholar and reference lists, (3) expert consultations. Additional databases could have been systematically searched. Experience from previous systematic search processes in such complex fields has shown that a combination of systematic searches and other methods is a good way to identify relevant articles [4]. However, with such a search procedure there is always a chance that something is left undiscovered. There is also a limitation associated with a single researcher conducting a study that may have affected the search and analysis process. Single studies with frameworks might have been overlooked [44] or specific biases in interpretation could have occurred in the analysis process [12]. These limitations were sought to be minimised through expert workshops and consultations to identify relevant frameworks and discuss the methodology and the analysis process of this article. Also, the exclusion criteria were very strict. Only literature published in the English language was included. The DNT related criteria were based on the knowledge of Krick et al. 2019 [2] and Krick et al. 2020 [4] but still led to a heterogeneity of frameworks due to the broadness of the field.

Only comprehensive, technology related frameworks were included, which ruled out many specific frameworks that could potentially also have been included. Frameworks focussing exclusively on special fields like health economics or acceptance could have been included, because they are certainly relevant for DNT, but were excluded due to their specificity and the large number of different frameworks available. Generalistic evaluation frameworks for (complex) healthcare interventions like the Consolidated Framework for Implementation Research (CFIR) [45] or on HTA Frameworks could have also been included (e.g. Integrate HTA [46]) – but it was necessary to limit the included frameworks to allow statements on this specific field of research.

The framework analysis itself has further limitations. Due to the heterogeneity of the frameworks and the difference in their content the categories and the assignment of the framework content to these categories is subjective. Even though the process of evaluation and classification was carried out with the utmost care, a non-overlapping categorization of these categories was not possible due to the complexity of the frameworks content and the interconnectedness of different categories. There are overlaps between the category of technology and individual as well as ethical and societal aspects. It should also be considered that the generalized statements made in this article can only provide initial indications in this specific field. No statistical analysis was carried out, but a qualitative and hypothesis-generating analysis. Despite these limitations, this article makes an important contribution to further research in the field of DNT evaluation.

## Conclusion

This research article provides orientation in the complex field of DNT evaluation. Eighteen relevant comprehensive evaluation frameworks for DNT have been identified. These frameworks focussed on different purposes and included various evaluation perspectives. The analysis and mapping in this article provide a good overview of the frameworks under consideration, their similarities and differences, evaluation areas, success definitions, strengths, and weaknesses. The assessment whether a DNT evaluation framework is good should be based on the clarity of the description of these aspects and the fulfilment of the quality criteria described.

The information on DNT evaluation frameworks provided in this review can therefore help in communication between decision makers and researchers to improve the evaluation process [11], by providing systematic information and a structure for the evaluation of a DNT. This can facilitate system implementation or provide helpful information in the technology development process. Comprehensive evaluation has the potential to avoid early system failures, prevent wrong investment decisions [10] or contribute to the development and implementation of better, more useful DNTs from a societal perspective. The general question what evaluation frameworks can contribute to this process should be further analyzed. The diversity and heterogeneity of frameworks presented in this article shows, that there is not one sole definition of the term “evaluation framework” in the field of DNT. The question of what constitutes a good DNT framework could also be further explored taking the criteria developed in this article as a starting point.

Future research could also address questions regarding what makes a successful DNT. The definition of a “successful” DNT, the role of the evaluation perspective and the purpose of the evaluation should be discussed when analyzing this question.

Overall, evaluators can use the concise information and quality criteria of this article as a starting point to select DNT evaluation frameworks for their research projects or to assess the quality of an evaluation framework for DNT, as well as a basis for exploring the open research questions raised.

## Supplementary Information

**Additional file 1.** Framework Analysis.

**Additional file 2.** Evaluation Areas.

**Additional file 3.** Detailed strength and weakness analysis.

## Data Availability

The datasets used and/or analyzed during the current study are included in this published article and the Additional files.

## Abbreviations

CFIR: Consolidated Framework for Implementation Research
CIS: Clinical Information Systems
CISSM: Clinical Information Systems Success Model
DHI: Digital Health Interventions
DIPSA: Development of an Evaluation Framework for Health Information Systems
DNT: Digital nursing technologies
EHR: Electronic Health Records
HIS: Health Information System
HTA: Health Technology Assessment
HITREF: Health Information Technology Evaluation Framework
HOPT-FIT: Human, Organization, Process and Technology-fit
IS: Information System
ICT: Information and Communication Technology
IHIS: Integrated Health Information Systems
MAST: Model for Assessment of Telemedicine
mHealth: Mobile health
NASSS: Nonadoption, abandonment, scale-up, spread, and sustainability
NCPF: Nursing care performance framework
RE-AIM: Reach, Effectiveness, Adoption, Implementation, and Maintenance
