# Supplementary material for: Evaluation frameworks for digital nursing technologies: analysis, assessment, and guidance. An overview of the literature
Source: BMC Nurs. 2021 Aug 17;20:146. doi: 10.1186/s12912-021-00654-8 (PMC8369663; doi:10.1186/s12912-021-00654-8)
Supplement: Supplementary file 1 — Additional file 1. Framework Analysis. [file 12912_2021_654_MOESM1_ESM.docx]

| **Framework** | **Top Category** | **Subcategories** | **Specification** |
| --- | --- | --- | --- |
| Nonadoption, abandonment, scale-up, spread, and sustainability Framework (NASSS) [1] | Condition | - Nature of condition or illness - Comorbidities, sociocultural influences | - What is the nature of the condition or illness? - What are the relevant sociocultural factors and comorbidities? |
|  | Technology | - Material features - Type of data generated - Knowledge needed to use - Technology supply model | - What are the key features of the technology? - What kind of knowledge does the technology bring into play? - What knowledge and/or support is required to use the technology? - What is the technology supply model? |
|  | Value Proposition | - Supply-side value (to developer) - Demand-side value (to patient) | - What is the developer’s business case for the technology (supply-side value)? - What is its desirability, efficacy, safety, and cost effectiveness (demand-side value)? |
|  | Adopters | - Staff (role, identity) - Patient (simple vs complex input) - Carers (available nature of input) | - What changes in staff roles, practices, and identities are implied? - What is expected of the patient (and/or immediate caregiver)—and is this achievable by, and acceptable   to, them?   - What is assumed about the ex- tended network of lay caregivers? |
|  | Organisation | - Capacity to innovate   (leadership etc.)   - Readiness for this technology (change) - Nature of adoption/   Funding decision   - Extend of change needed to routines - Work needed to implement change | - What is the organisation’s capacity to innovate? - How ready is the organisation for this technology-supported change? - How easy will the adoption and   funding decision be?   - What changes will be needed   in team interactions and routines?   - What work is involved in implementation   and who will do it? |
|  | Wider System | - Political/ policy - Regulatory/ legal - Professional - Socio-cultural | - What is the political, economic, regulatory, professional (eg, medicolegal), and sociocultural context for program rollout? |
|  | Embedding and adaption over time | - Scope for adaption over time - Organisational resilience | - How much scope is there for adapting and coevolving the technology and the service over time? - How resilient is the organisation to handling critical events and adapting to unforeseen eventualities? |
| Model for Assessment of Telemedicine (MAST Manual) [2] | Preceding consideration | - Purpose of the telemedicine application? - Relevant alternatives? - International, national, regional or local level of assessment? - Maturity of the application |  |
|  | Health problem and characteristics of the application | - Health problem - Description of the application - Technical characteristics | - Definition of target condition/disease - Symptoms, consequences - Number of patients (epidemiology) - Burden of disease, resource use - Current management of health condition - Existing quality standards - Relations to other conditions or treatments. (Does the service have implications for treatment of competing   disease)   - Change in patient segments (will the service increase or decrease the group of patients who can benefit from or   will get the service offered)   - Features of the application - Tools required for using the application - Training and information needed for utilizing the   application (staff and patients)   - Maturity of the telemedicine application (life cycle) - Division of responsibility for the technical solution   between involved organisations.   - Regulatory status - Technical platform - Market situation - Infrastructure requirements - Interoperability: Integration needs (EPR, devices, with current applications, technical standards etc.) - Technical support - Technical environment - Standard situation - User support - Back-up systems and procedures |
|  | Safety | - Clinical safety (patients and staff) - Technical safety (technical reliability) | - What are the direct or indirect harms when using the telemedicine application? - What is the scope of the harms? - What are the types of harms? - Are there estimates of incidence of harms? - What is the timing of onset of harms? - What is the duration and severity of the harms? - What can be done to minimise the harms? - Is there a backup system and how does it work? - What do the Service Level Agreements cover? - Does the technology experience interference and what are the consequences? - How is the safety compared to alternative technologies? - How is security of data and the database (data privacy) and quality of data managed?   - encryption/cryptography   - data storage and ownership   - data ownership |
|  | Clinical effectiveness | - Effects on mortality - Effects on morbidity - Physical health - Mental health - Effects on health related quality of life (HRQL) - Behavioural outcomes (e.g. exercise) - Utilization of health services |  |
|  | Patient perspectives | - Satisfaction and acceptance - Understanding of information - Confidence (in the treatment) - Ability to use the application - Access and accessibility - Empowerment, self-efficacy |  |
|  | Economic aspects | - Economic evaluation (societal perspective) - Business case (institutional level) - Sensitivity analysis (Risk analysis) | - Amounts of resources used when delivering the assessed telemedicine application and its comparators in the health care sector and other sectors (e.g.):   - Investments in equipment   - Training of staff   - Maintenance - Unit costs or prices for each resource used - Related changes in use of health care resources (e.g.):   - Primary care   - Emergency unit - Expenditures per year (including expenditures related to the resource use described in the cost estimation above) - Revenue per year:   - Activity (number of patients or services)   - Reimbursement (e.g. DRG-rate) per service or patient |
|  | Organisational aspects | - Process - Structure - Culture - Management | - Workflow - Staff, training and resources - Interaction and communication - Spread of technology, centralization or decentralization - Economy (see domain on economic aspects) - Attitude and culture |
|  | Socio-cultural, ethical and legal aspects | - Ethical issues - Legal issues - Social issues | - Overall questions: Does the application challenge religious,   cultural or moral beliefs?   - Potential ethical problems, e.g. giving the responsibility to the Patients - Autonomy: Is the patient’s autonomy challenged or increased? - Equity - Clinical accreditation - Information governance - Professional liability - Patient control – consent, access - Changes in the patients role in major life areas (e.g. social life,   working life)   - Patients’ relatives and others’ understanding of the technology - Societal, political context and changes. Will the service influences   the general model for the delivery of healthcare service if deployed   - Changes in responsibility. Are the patients and/or relatives capable of handling their responsibility? - Gender issues. Has the service any consequences on the position of gender? |
|  | Transferability Assessment | - Cross-border - Scalability - Generalizability |  |
| Infoway benefits evaluation Framework [3] | System Quality | - Functionality - Performance - Security | - Type of feature and level of decision support - Accessibility (distance and availability) reliability (down time, system response time) - Type of features |
|  | Information Quality | - Content - Availability | - Completeness, accuracy, relevance, comprehension - Timeliness, reliability and consistency of information when and where needed |
|  | Service Quality | - Responsiveness | - User training, ongoing technical support and availability of support |
|  | Use | - Use/behaviour pattern - Self-reported use - Intention to use | - Frequency, duration, location, type or nature and flexibility of actual usage - Frequency, duration, location, type or nature and flexibility of perceived usage - Proportion of and factors for current non-user to become user |
|  | User Satisfaction | - Competency - User Satisfaction - Ease of use | - Knowledge, skills and experience - Perceived expectations, value, information/system/service quality and use of the system (including provider – patient interaction, preference, comfort and experience) - User friendliness and learnability |
|  | Net Benefits | - Quality - Access - Productivity | - Patient Safety - Appropriateness/effectiveness - Health outcomes - Ability of patient/provider to access service - Patient and caregiver participation - Efficiency - Care coordination - Net Cost |
|  | Organisational Context Factors | - Strategy - Culture - Business Process |  |
| Health Information Technology Evaluation Framework (HITREF) [4] | Structural Quality | - Organizational Support/ Capacity - Hardware - Software - Functionality |  |
|  | Quality of Information  Logistics | - Completeness/   Correctness of data   - Costs of information processing - User satisfaction - Patient privacy - Patient satisfaction with HIT - Diffusion |  |
|  | Unintended Consequences/  Benefits | - Unintended Consequences/   Benefits |  |
|  | Effects on Outcome Quality of  Care | - Patient outcome - Costs of patient care - Patient satisfaction   with care   - Patient related   knowledge |  |
|  | Effects on Quality Processes | - Efficiency - Appropriateness of patient care - Organizational or social   Quality   - Clinical involvement in HIT Selection, Implementation, Training |  |
|  | Barriers or Facilitators to Adoption | - Barriers or Facilitators to   Adoption |  |
| Evaluation Framework for Fit-For-Purpose Connected Sensor Technologies [5] | Verification, analytical validation, and clinical validation |  | - Does the tool measure what it claims to measure? - Is the measurement appropriate for the target population? |
|  | Security practices |  | - Does the manufacturer build with safety by design? Is there a disclosure policy? Software bill of materials? |
|  | Data rights and governance |  | - Who has access to the data and when? Is the privacy policy publicly accessible? |
|  | Utility and usability |  | - How is the tool worn? Battery life? Available technical support? |
|  | Economic feasibility |  | - What’s the net benefit vs price? Is cost a one-time or a subscription model? |
| RE-AIM (Reach, Effectiveness, Adoption, Implementation, and Maintenance.)  (expanded to clinical informatics) [6] |  |  |  |
|  | Reach  (individual level) | - Absolute number - proportion - representativeness   of individuals who are willing  to participate in a given  initiative, intervention, or  program | - What percentage of the target population came into contact with or began program? - Did program reach those most in need? - Were participants representative of your practice setting? |
|  | Efficacy/effectiveness  (individual level) | Impact of an intervention on:   - Important outcomes - Potential negative effects - Quality of life - Economic outcomes | - Did program achieve key targeted outcomes? - Did it produce unintended adverse consequences? - How did it affect quality of life? - What did the program cost as implemented and what would it cost in your setting? |
|  | Adoption (setting  and/or organizational  level) | - Absolute number - proportion, - representativeness   of settings and intervention agents (people who deliver  the program) who are willing to initiate a program | - Did low-resource organizations serving high-risk populations use it? - Did program help the organization address its primary mission? - Is program consistent with your values and priorities? |
|  | Implementation  (setting and/or organizational level) | Intervention agents’ fidelity to the various elements of an intervention’s protocol, including consistency of delivery as intended and  the time and cost of the intervention; individual level—clients’ use of the intervention strategies | - How many staff members delivered? - Did different levels of staff implement the program successfully? - Were different program components delivered as intended? |
|  | Maintenance  (individual and setting and/or organizational  levels) | Extent to which a program or policy becomes institutionalized or part of the routine organizational practices and policies; individual level—long-term effects of a program on outcomes for 6 or more month after the most recent intervention contact | - Did program produce lasting effects at individual level? - Did organizations sustain the program over time? - How did the program evolve? - Did those persons and settings that showed   maintenance include those most in need? |
| Adapted nursing care performance framework [7] | Acquiring, Deploying, and Maintaining Resources | - Time and Efficiency | - Time Management - Time Spent for Patient Care - Documentation Time |
|  | Transforming Resources into Services | - Nurses’ Practice Environment - Nursing Processes - Professional Satisfaction | - Knowledge Updating and Utilization - Information Quality and Access - Nurse Autonomy - Intra- and Interprofessional Collaboration - Nurses’ Competencies and Skills - Quality of Documentation - Nurse-Patient Relationship - Assessment, Care Planning, and Evaluation - Teaching of Patients and Families - Communication and Care Coordination - Nurses’ Perspectives of the Quality of Care Provided - Satisfaction or Dissatisfaction of Nurses Using ICTs |
|  | Producing Changes in Patients’ Condition | - Nursing-Sensitive Outcomes | - Patient Comfort and Quality of Life Related to Care - Empowerment - Functional Status - Satisfaction or Dissatisfaction of Patients of Using ICTs |
| Design and Evaluation  of DHI Framework)[8] | Ease of use  Content Quality  Privacy & security  Accountability  Adherence  Aesthetics  Perceived benefit  Effectiveness  Service Quality  Personalization  Perceived enjoyment  Ethics  Safety |  | - The degree to which effort is required to take   advantage of the DHI (e. g., using common  interaction paradigms).   - The degree to which the content of a DHI is accurate, timely, complete, relevant, and   consistent (e. g., real-time location-based  pollen warnings for asthmatics)   - The degree to which the DHI considers legal   requirements and aspects with respect to  privacy and security aspects (e. g., a DHI is  compliant with the General Data Protection  Regulation).   - The degree to which information about the   DHI is made explicit for usage decisions  (e. g., details of the intervention author of a  DHI are accessible).   - The ratio of actual usage to intended usage   of a DHI (e. g., 4 out of 5 exercises are  conducted per week).   - The degree to which the DHI interface   applies design elements, colors and fonts in  a logical way (e. g., consistent use of colors,  figures and fonts).   - The degree to which a person believes that   using a DHI improves his or her health behavior/health condition (e. g., a believe  that a DHI helps to increase physical activity)   - The degree to which the DHI contributes to   the enhancement of an individual’s health  behavior/condition (e. g., significant  reduction of fat mass).   - The extent to which support of a DHI is   provided (e. g., a technical support line is  made available).   - The degree to which the DHI adapts to the   needs of an individual (e. g., the daily step  goal of a DHI adapts to the capabilities of an  individual).   - The degree to which an individual believes   that using a DHI is engaging (e. g., the use of  game elements and level designs in a DHI).   - The degree to which the DHI addresses   ethical aspects (e. g., the DHI was designed  for individuals with various cultural  backgrounds or disabilities).   - The extent to which the usage of a DHI is safe with respect to side effects (e. g., interactions with a DHI are limited to account for addiction behavior |
| Health technology assessment framework for digital healthcare services (Digi HTA) [9] | Company information  Product information  Technical stability  Cost  Effectiveness  Clinical Safety  Data security and protection  Usability and accessibility  Interoperabilty  Artificial intelligence  Robotics |  | - Contact information of company. - What is the company’s business model? - Are quality management systems in use? Which ones? - The name of the product. - Short description of the product. - What is the product’s readiness level (TRL levels 1–9)? - Which platforms and platform versions of the product are available? - Does the product have CE and/or FDA approval? - Is the product a medical device, and what classification does it have? - Is the product classified according to MDD or MDR requirements? - Does the product meet the electrical safety requirements for medical devices (if applicable)? - Does the use of the product require registration or login? - Does the use of the product require strong identification? - Does the company have any plans for post-market surveillance of the product? - What kind of product support does the company offer? - What is the intended use of the product? - What are the intended user groups? - What problem in the healthcare system is the product trying to solve? - Is the aim of the product to replace any existing healthcare services? - Does the introduction of the product cause any changes to the premises, information systems, or care processes? - Is the product already in use elsewhere in Finland or worldwide? - Where, and for how long? - What kind of support does the end user need to use the product? - If users need training, who organizes it? When?   What is the language of training?   - Does the company have instructions (e.g., a project plan) for healthcare service providers to ensure fluent introduction of the product? - What is the company’s testing process? - What is the company’s process for handling error messages? - Does the company have the capacity to roll back to previous versions of the product? - Does the company have a process to proactively monitor the running of systems and system components to automatically identify faults and technical issues? - Does the company have a plan for decommissioning the product? - Has there been any downtime or impairment time in the use of the product during the last six months? - What are the costs of using the product for a healthcare customer? - If the use of the product is free, what is the source of the company’s income? - What kind of initial costs (estimated minimum and maximum values in detail) does the introduc-tion of the product impose on the organization, including changes to buildings or facilities, a need for new devices and software, as well as needed training? - What are the maintenance costs (estimated minimum and maximum values) to the organization for the use of the product? - How often must devices or software versions related to the product be renewed? - Which uncertainties apply to these cost estimates? - Does the product provide clinical benefits? What are they? - Does the product provide benefits to the end users by improving their behavior related to their own health? How so? - Does the product provide benefits to the organization (like improving care processes)? How so? - What kind of evidence is available for effectiveness (case studies, randomized controlled trials, Cochrane reviews, etc.)? - Are there any ongoing studies to investigate the product’s effectiveness? - Does any institution like the Duodecim Current Care Guidelines recommend the use of the product? - Are there any risks, possible side effects, or other undesirable effects associated with using the product? - Is there any research evidence available related to clinical safety? - Have any product-related adverse events been reported or identified? - What is the company’s process to handle adverse events? - Has the product undergone a risk analysis? - Are there any undesirable effects associated with misuse of the product? - Are the error conditions of guidelines removed, or is their realization unlikely? - Is the company aware of the product register and Manufacturer Incident Report supervised by the National Supervisory Authority of Welfare and Health? - Who is the responsible person in the company for handling Manufacturer Incident Reports? - Data Security and Protection Preliminary Task - Information Security and Data Protection Requirements - Have all user groups been taken into account in product design, like people with visual or hearing impairments? - Has the product been tested with real user groups? - What kind of accessibility testing has been performed on the product? - Has the functionality of the product been tested with screen readers or other assistive technol-ogies? - How have the product’s users been taken into account in the product’s text (clear, concrete language; the avoidance of professional language)? - How have the product’s users been taken into account in the design of its textual content (headings, lists, and images)? - How does the company continue to collect feedback from users and make changes to the product based on this feedback? - What changes have been made to the product based on user feedback? - How is the company going to continue to evaluate and develop accessibility? - Is the product compatible with the following ty guidelines (if applicable)?   WCAG 2.0/ WCAG 2.1  Papunet Design Guide for Websites  EN 301 549 section 11-Software  Design guidelines for native application  Design guidelines for progressive web application   - Does the application support OS accessibility features? - Does the product have interfaces into the website or other software? - Does the product have interfaces into the following healthcare services? Electronic patient records (which ones?) Finnish Kanta PHR Other (what?) - Are proprietary formats used to store and transfer data? - Are the definitions of the original proprietary formats openly available? - Does the product have interfaces for other companies’ services? - Can the data contained in the product be exported in a commonly used or standard format? - Does the product use data from other systems via interfaces? If yes, can the data produced by others be separated in the system? - Does the product connect with health or wellness devices? If yes, is it compatible with ISO/IEEE 11073 Personal Health Data (PHD) Standards? - Exactly what defined problem is going to be solved by the AI? - What is the classification of AI? Visualization only, AI–assisted (e.g., diagno-sis/classification/decision), or solely AI–controlled? - Could the problem be solved without the AI solution? - Is the solution based on machine learning or a neural network? - Do the staff have sufficient capacity to understand the operational logic of AI (e.g., do they need additional training)? - Are the conclusions and decisions of the AI solution transparent, i.e., can medical staff understand what the decisions are based on? - Is the AI solution validated in the environment in which it will be used? - What are the data sources for the AI solution? - Are the data sources used in the training of AI solutions relevant to a final use case (e.g. are the age and gender composition of training groups comparable to that of real user groups)? - Are the access rights required for the use of the data in order, and have data protection (e.g., GDPR) and security issues been taken into account? - When it comes to classifier teaching, are there enough data relative to the size of the smallest class? - Can the AI solution use incomplete data? Can the AI solution use noisy data? - Is retraining possible for the AI solution? What are the data sources for retraining? - How is it ensured that the system is not taught with irrelevant data? - How many tests or results are needed for the AI model? - Is the algorithm purchased software as a service (SaaS) or its own design? What performance criteria are used? - Does the AI solution change care processes? How? - When does the AI solution propose an action? - How, and who will actually implement it? - Is staff’s approval needed for action proposed by the AI? - Is there any possibility that using the robot could create safety risks for healthcare personnel or customers (e.g., forces that could be destructive or collision with people)? - How have those risks been avoided in the robot’s design? - What kind of arrangements are needed to teach or program the robot to operate? - If the robot is battery-operated, what are the operating, idle, and charging times? |
| Clinical Information Systems Success Model (CISSM) [10] | System Performance (SP)  Information Quality (IQ)  Social Influence (SI)  Facilitating Conditions (FC)  CIS Use Dependency  Nurse Satisfaction  Net Benefit | - Ease of Use - Accessibility - Reliability - Perceived Usefulness - Content Completeness - Format - Accuracy - Service Support - Social Support - Perceived Behavioral Control - Work Processes | - The degree to which a person believes that using a particular system would be free of effort - The ease or difficulty with which the user may act to utilize the capability of the computer system - The dependability and consistency of access and uptime of systems - The degree to which a person believes that using a particular system would enhance his or her job performance - The completeness and precision of the output information - The material design of the layout and display of the output contents - The correctness of the output information - The degree to which clinicians perceive a helpful network of   co-workers   - User’s expectations and perceptions of service performance levels provided by CIS staff - Perceptions of internal and external constraints on behavior - The clinician’s perceptions of clinical activities as they relate to specific CIS applications - The extent to which the CIS is integrated into the clinician’s work routine - The level of overall clinician satisfaction with CIS - The degree to which a clinician believes that using a particular CIS   impacts job performance |
| Comprehensive evaluation framework for telemedicine implementation [11] | Human | - Service Provider - Patient/clinic | - Comfort with   Work flow   - Expertise on ICT - Education &   Training   - Comfort with   Patient communication   - Comfort with Provider interaction - Resistance to   Change   - Location/ travel time - Disease characteristics - ICT skills & knowledge - Patient awareness - ICT equipment - User habit - Medical cost   (out-of-pocket) |
|  | System | - Organisation - Technology | - Leadership - Organizational culture - Change management - Hospital information   systems   - Budget - Training & support - Work flow reengineering - Reliability of technology - Storage - System speed - User interface - Data quality - Transmission - Interoperability - Information security |
|  | Environment | - Society - Rules/policy | - Reimbursement - 3rd party payer - Insurance fee   schedule   - ICT infrastructure - Social norms & values - Governmental authority - Interface standards - Privacy certification &   license   - Privacy & security rule - Practice Medical liability |
|  | Outcomes | - Cost effectiveness - Quality of care - Patient satisfaction |  |
| Digital Health Score Card [12] | Technical    Clinical  Usability  Cost | - Performance - Security - Interoperability - Evidence - Usability (helpful, learnable, likable) - Purchasing price - Resources - Anticipated costs | - Assessment of performance when compared to technical gold standard - Testing of security - Testing of interoperability - Critical appraisal of evidence supporting   whether solution has impact on defined clinical  outcome   - Comparison to existing clinical gold standard - Real world testing or simulation performance intarget population - Assessment using standardized usability   framework that evaluates  Performance across basic  Characteristics (e.g. helpful; effective; learnable; likeable)   - Purchase price - Resources including time   required for training, set-up, implementation,  and management  of solution   - Anticipated cost impact on clinical outcome of interest |
| Development of an Evaluation Framework for Health Information Systems (DIPSA Framework) [13] | Technology  Human Factor  Organization | - System quality - Safety - Collaboration - Satisfaction - Procedures |  |
| Health Technology Adoption Framework [14] | Health gain | - Efficacy (evidence based   medicine, clinical outcomes and quality of life)   - Population health (burden of disease) - Standard of care | - Is there evidence that the technology will improve individual patient short-term (, 5 years) gain in health (clinical outcomes and/or quality of life) as   compared with the current practice?   - Is there evidence that the technology will improve individual patient long-term (5 years) gain in health or reduce the   likelihood of further disease or complications as compared with the  current practice?   - Can the technology, including risk of adverse events, benefit cases with few alternatives? - Does the technology address a condition with significant incidence and/or prevalence (burden of disease)? - Is the incidence or prevalence projected to increase or decrease over the next 5   years?   - Has the technology become the Standard of Care in other health regions? - Will the technology establish a new Standard of Care? |
|  | Service delivery | - Safety - Training - Access - Service coordination - Sustainability | - Is the technology at least as safe as current practice for the patients? - Is the technology at least as safe as current practice for the health care providers? - Will the technology require health care provider training? - What is the expected time frame for more health care providers to acquire the expertise to use the technology? - Will the technology improve accessibility (i.e. shift services closer to where patients reside; geographic equity)? - Will the technology provide services to under-served population(s)? - Will the technology improve the provision of services at the most appropriate time or decrease wait times? (Timeliness; service efficiency)? - Will the technology improve coordination and collaboration with other clinical services or reduce or increase impact on other services (service coordination)? - Will the technology reduce load or positively impact other services? - How many health care providers are demanding this technology? - Will the technology be well utilized? - How many health care providers have the expertise to use the technology upon acquisition? - Will additional human resources be required? |
|  | Strategic fit | - Strategic fit | - Is the technology aligned with internal (Department/Division) strategic goals? |
|  | Innovation | - Knowledge and research | - Will the technology improve the generation, transfer, and/or application of new knowledge to patient care services? (innovation characteristics) |
|  | Financial | - Cost (resources, infrastructure) - Economic analysis (cost-effectiveness,   cost-benefit) | - Will the technology have Direct costs (purchase of technology)? - Will the technology have One Time and Start Up Costs? - Will the technology have Ongoing costs? - Will the technology impact Other Services Areas? - Will the technology have Alternative or Partial Funding Sources? - Will the technology have   Environmental costs?   - Is there evidence to support the cost-effectiveness of the technology? - Is the cost-effectiveness threshold the same for all (e.g. children vs adults)? - Is there evidence to support the cost-benefit   ratio of the technology?   - Are any potential cost increases associated with the technology offset by significant improvements in quality of life or other patient outcomes? |
| Hospital Information SystemSuccess Framework [15] | Functional | - Preparation of the user requirements - Alignment of the role and design of the HIS (Task-technology adaption) - Flexibility towards dynamic changes and changes in the organizational context - Added functionality are provided by the HIS, enabling users to provide new or better services - Improve clinical performance and outcomes - In general |  |
|  | Organizational | - Collaboration and cooperation - Participation in decision-making - Work from the workflow - Support from higher level organizations - Make implementation a transparent process within the organization - Organizational stability - Rate of hospital independence and authority - Organizational capacity for changes - In general |  |
|  | Behavioral | - User involvement - User engagement and commitment - Resistance to changes - User knowledge and skills - Stakeholder, user and patient satisfaction - Motivational activities - User acceptance (perceived system ease of use, perceived system usefulness) - In general |  |
|  | Cultural | - Understand health care as a specific culture - Understand the local culture (such as attention to cultural differences between public and private hospitals as well as developing and developed countries) - Preparedness and willingness towards cultural change (professional culture) - Expectations of users - In general |  |
|  | Management | - Managers commitment - Formulation and expression of a clear vision for the enterprise   showing the HIS as part of it   - Setting clear goals and instructions - Flexible planning - Prospective and proactive control - Coping with the impact of change - Internal communication and clear feedback - Having a strategy - Handling the diversity within stakeholder goals - Using formal project management methodology - Dedicate, availability and prioritize of competitive hospital   resources (human, financial and physical resources and time)   - Identify and mitigate risk (risk management) - Consider IT implementation as a change process 1 - Understanding socio-technical nature of HIS - Regular evaluations and using their results at different stages of HIS life cycle - In general |  |
|  | Technical (system  quality, information  quality and service  quality) | - Integration with Legacy system - Interoperability and Interconnectivity - Usability - Balance between flexibility and stability of IT - Reliable technical infrastructure or network - Complexity of the system - Information quality (relevancy, usefulness, completeness, etc.) - Response time (system speed) - System security - Service quality (the support provided by the information department, the support provided by the maintenance company) - Quality of user documentation - Flexibility and adoptability, enabling future functional and technical changes - Using proper standards, coding and nomenclature - In general |  |
|  | Strategy | - National, regional, organizational - Accepted also at lower levels - Alignment between system strategies and hospital strategies - In general |  |
|  | Economy | - Return on investment (material or immaterial) - Justification of increase of costs - Sufficient funding - In general |  |
|  | Education | - Sufficient training to make the best out of the daily operation - Sufficient training to provide an understanding of its limitations and future potentials - In general |  |
|  | Legal | - Compliance with legal requirements - Know what the legal constraints/opportunities - In general |  |
|  | Ethical | - Compliance with existing ethical rules in affairs management - Privacy and confidentiality - In general |  |
|  | Political | - Political games/conflicts - Willingness towards investment on IT systems - Reliable external partners - In general |  |
| Human, Organization, Process and Technology-fit (HOPT-FIT) [16] | Human  Organisation  Process  Technology | - System Design - System Implementation - System Use - Structure - Environment - Clinical Flow/Standard - Business Process Management - Lean Method - System Quality - Information Quality - Service Quality |  |
| Khoja–Durrani–Scott Evaluation Framework [17] | Health services outcomes  Technology outcomes  Economic outcomes  Behavioral and  sociotechnical outcomes   - Ethical outcomes   Readiness and change outcomes  Policy outcomes | - Development - Implementation - Integration - Sustained Operation - Development - Implementation - Integration - Sustained Operation - Development - Implementation - Integration - Sustained Operation - Development - Implementation - Integration - Sustained Operation - Development - Implementation - Integration - Sustained Operation - Development - Implementation - Integration - Sustained Operation - Development - Implementation - Integration - Sustained Operation | - Ongoing and periodic assessment of health status, existing services,   needs, and opportunities   - Improved diagnosis and treatment of disease conditions - Improved decision support and clinical   care and health management   - Improved access to care - Barriers and facilitators - Acceptability of e-health - Better clinical safety - Improved quality of care - Functional independence among staff - Equity of care - Stability of services - Effects on the delivery of medical care - Health impact leading to   change in disease status   - Social impact due to improved access and quality of services - Stability of services - Improvement in quality of life - Health impact showing change via indicators - Stability of services - Wide reach - Development cost, availability, affordability - Interoperability and standardization - Well-designed software - Reliable hardware - Technical efficiency or fix - Timeliness - Cost - Robust and reliable networking - Easily adaptable to different settings (patenting) - Cultural acceptability - Environmental viability - Interoperability - User-friendliness/usability - Appropriate in a variety of conditions - Relevance to existing and growing needs - Flexible (can be modified to suit local cultural/social needs) - Efficiency/error rates - Accuracy - User acceptance - Appropriate in a variety of conditions - Relevant to existing and growing needs - Broader interoperability - Scalability - Cost benefit - Ability to be incorporated   into policy   - Affordability - Cost minimization - Cost-utility - Cost-benefit - Improved DALYs - Improved QALYs - Human resource factors (management style, working relationship,   communications flow, staff motivation)   - Strategy for e-health implementation - User-friendliness - Human–computer interaction - Direct benefits to users in routine work - Benefits in learning - Penetration/diffusion of innovation (addressing the digital divide) - Trust - Beneficence/nonmaleficence (client, provider, organization) - Problem handling - Gender issue/gender divide - Penetration/diffusion of   innovation (addressing  the digital divide)   - Strategy for broader e-health adoption - Adoption/adaptation of technology on a wider   Scale   - Prioritizing e-health over other issues - Moral consideration - Autonomy (client based) - Justice and equity - Selection of study subjects/patients and population - Securing identity and maintaining confidentiality of patient information - Sensitive to sociocultural issues - Security - Liability - Licensure - Reimbursement - All of the following in a   broader perspective:   - Sensitive to sociocultural   issues   - Security - Liability - Licensure - Reimbursement - Security - Plan for change management - Individual, organizational, and societal readiness to technology change - "Involvement" of end user in requirements elicitation phase, selection of vendor, solution, evaluation, features, etc. - Effective change management   (preparation and action)   - Training of all staff, including clinical and management staff - Effective change management (maintenance) - Modification - Improvement - Customization - Policies for change management - Scope for innovations - Funding support for research - Limited changes in organizational and national policies to facilitate e-health implementation - Policy changes to   facilitate broader  adoption, implementation,  and innovation in  e-health   - Healthy public policy and   organizational practice   - Knowledge sharing with   other organizations and  countries |
| The layered telemedicine implementation model [18] | Technical  Behavioural  Economical  Organizational | - Technology - Acceptance - Financial - Organization - Policy and Legislation | - Support - Training - Usability - Quality - Attitude and usability - Evidence based medicine - Diffusion and dissemination - Provider and structure - Intramural and extramural work practices - Legislation and policy - Standardization - Security |

1. Greenhalgh T, Wherton J, Papoutsi C, et al. (2017) Beyond Adoption: A New Framework for Theorizing and Evaluating Nonadoption, Abandonment, and Challenges to the Scale-Up, Spread, and Sustainability of Health and Care Technologies. J Med Internet Res 19:e367

2. Kidhom K, Bowes A, Dyrehauge S, et al. (2010) The MAST Manual. MAST - Model for ASsessment of Telemedicine. In:MethoTelemed team

3. Francis Lau F, Hagens S, Muttitt S (2007) A Proposed Benefits Evaluation Framework for Health Information Systems in Canada. Healthcare Quarterly 10

4. Sockolow PS, Crawford PR, Lehmann HP (2012) Health services research evaluation principles. Broadening a general framework for evaluating health information technology. Methods Inf Med 51:122-130

5. Coravos A, Doerr M, Goldsack J, et al. (2020) Modernizing and designing evaluation frameworks for connected sensor technologies in medicine. npj Digital Medicine 3:37

6. Bakken S, Ruland CM (2009) Translating clinical informatics interventions into routine clinical care: how can the RE-AIM framework help? Journal of the American Medical Informatics Association : JAMIA 16:889-897

7. Rouleau G, Gagnon MP, Côté J, Payne-Gagnon J, Hudson E, Dubois CA (2017) Impact of information and communication technologies on nursing care: Results of an overview of systematic reviews. Journal of Medical Internet Research 19

8. Kowatsch T, Otto L, Harperink S, Cotti A, Schlieter H (2019) A design and evaluation framework for digital health interventions. it - Information Technology

9. Jari H, Niina K, Petra F, Anna M, Timo K, Jarmo R (2019) Digi-HTA: Health technology assessment framework for digital healthcare services. Finnish Journal of eHealth and eWelfare 11

10. Garcia-Smith D, Effken JA (2013) Development and initial evaluation of the Clinical Information Systems Success Model (CISSM). Int J Med Inform 82:539-552

11. Chang H (2015) Evaluation Framework for Telemedicine Using the Logical Framework Approach and a Fishbone Diagram. Healthcare informatics research 21:230-238

12. Mathews SC, McShea MJ, Hanley CL, Ravitz A, Labrique AB, Cohen AB (2019) Digital health: a path to validation. NPJ Digit Med 2:38

13. Stylianides A, Mantas J, Roupa Z, Yamasaki EN (2018) Development of an Evaluation Framework for Health Information Systems (DIPSA). Acta informatica medica : AIM : journal of the Society for Medical Informatics of Bosnia & Herzegovina : casopis Drustva za medicinsku informatiku BiH 26:230-234

14. Poulin P, Austen L, Scott CM, et al. (2013) Multi-criteria development and incorporation into decision tools for health technology adoption. Journal of health organization and management 27:246-265

15. Sadoughi F, Kimiafar K, Ahmadi M, Shakeri MT (2013) Determining of factors influencing the success and failure of hospital information system and their evaluation methods: A systematic review. Iranian Red Crescent Medical Journal 15

16. Yusof MM (2019) A Socio-Technical and Lean Approach Towards a Framework for Health Information Systems-Induced Error. Stud Health Technol Inform 257:508-512

17. Khoja S, Durrani H, Scott RE, Sajwani A, Piryani U (2013) Conceptual framework for development of comprehensive e-health evaluation tool. Telemed J E Health 19:48-53

18. Broens T, Veld R, Vollenbroek - Hutten M, Hermens H, Halteren A, Nieuwenhuis B (2007) Determinants of successful telemedicine implementations: A literature study. Journal of telemedicine and telecare 13:303-309
