# Supplementary material for: Evaluation frameworks for digital nursing technologies: analysis, assessment, and guidance. An overview of the literature
Source: BMC Nurs. 2021 Aug 17;20:146. doi: 10.1186/s12912-021-00654-8 (PMC8369663; doi:10.1186/s12912-021-00654-8)
Supplement: Supplementary file 2 — Additional file 2. Evaluation Areas. [file 12912_2021_654_MOESM2_ESM.docx]

| **Canvas Category** | **Framework Category** | **Related Framework** |
| --- | --- | --- |
| **Focus** | Nature of condition or illness | Nonadoption, abandonment, scale-up, spread, and sustainability Framework (NASSS) |
|  | Health problem | Model for Assessment of Telemedicine (Mast Manual) |
|  | Disease characteristics | Comprehensive evaluation framework for telemedicine implementation |
|  | Comorbidities | Nonadoption, abandonment, scale-up, spread, and sustainability Framework (NASSS) |
|  | Purpose of the telemedicine application | Model for Assessment of Telemedicine (Mast Manual) |
|  | Relevance to existing and growing needs | Khoja–Durrani–Scott Evaluation Framework |
| **Product/**  **Technology** | Material features | Nonadoption, abandonment, scale-up, spread, and sustainability Framework (NASSS) |
|  | Aesthetics | Design and Evaluation of DHI Framework |
|  | User interface | Comprehensive evaluation framework for telemedicine implementation |
|  | System Design | Human, Organization, Process and Technology-fit (HOPT-FIT) |
|  | Well-designed software | Khoja–Durrani–Scott Evaluation Framework |
|  | Format (material design of the layout and display) | Clinical Information Systems Success Model (CISSM) |
|  | Alignment of the role and design of the HIS (Task-technology adaption) | Hospital Information System Success Framework |
|  | Technology supply model | Nonadoption, abandonment, scale-up, spread, and sustainability Framework (NASSS) |
|  | Maturity of the application | Model for Assessment of Telemedicine (Mast Manual) |
|  | Functionality | Infoway benefits evaluation Framework |
|  | Performance | Infoway benefits evaluation Framework |
|  | Ease of use | Infoway benefits evaluation Framework |
|  | Responsiveness | Infoway benefits evaluation Framework |
|  | Software (Usability) | Health Information Technology Evaluation Framework (HITREF) |
|  | Functionality | Health Information Technology Evaluation Framework (HITREF) |
|  | Usability | Evaluation Framework for Fit-For-Purpose Connected Sensor Technologies |
|  | Ease of use | Design and Evaluation of DHI Framework |
|  | Technical stability | Health technology assessment framework for digital healthcare services (Digi HTA) |
|  | Usability | Health technology assessment framework for digital healthcare services (Digi HTA) |
|  | Ease of Use | Clinical Information Systems Success Model (CISSM) |
|  | Reliability | Clinical Information Systems Success Model (CISSM) |
|  | Accuracy | Clinical Information Systems Success Model (CISSM) |
|  | Reliability of technology | Comprehensive evaluation framework for telemedicine implementation |
|  | Storage | Comprehensive evaluation framework for telemedicine implementation |
|  | System speed | Comprehensive evaluation framework for telemedicine implementation |
|  | Transmission | Comprehensive evaluation framework for telemedicine implementation |
|  | Performance | Digital Health Score Card |
|  | Usability (helpful, learnable, likable) | Digital Health Score Card |
|  | System quality | Development of an Evaluation Framework for Health Information Systems (DIPSA Framework |
|  | Preparation of the user requirements | Hospital Information System Success Framework |
|  | Usability | Hospital Information System Success Framework |
|  | Balance between flexibility and stability of IT | Hospital Information System Success Framework |
|  | Reliable technical infrastructure or network | Hospital Information System Success Framework |
|  | Complexity of the system | Hospital Information System Success Framework |
|  | Response time (system speed) | Hospital Information System Success Framework |
|  | Flexibility and adoptability, enabling future functional and technical changes | Hospital Information System Success Framework |
|  | System Quality (measures of the information processing system itself) | Human, Organization, Process and Technology-fit (HOPT-FIT) |
|  | Reliable hardware | Khoja–Durrani–Scott Evaluation Framework |
|  | Technical efficiency or fix | Khoja–Durrani–Scott Evaluation Framework |
|  | Robust and reliable networking | Khoja–Durrani–Scott Evaluation Framework |
|  | User-friendliness/usability | Khoja–Durrani–Scott Evaluation Framework |
|  | Usability | The layered telemedicine implementation model |
|  | Quality | The layered telemedicine implementation model |
|  | Type of data generated Data | Nonadoption, abandonment, scale-up, spread, and sustainability Framework (NASSS) |
|  | Description of the application | Model for Assessment of Telemedicine (Mast Manual) |
|  | Content | Infoway benefits evaluation Framework |
|  | Completeness/Correctness of data | Health Information Technology Evaluation Framework (HITREF) |
|  | Content quality (accurate, timely, complete, relevant, and consistent) | Design and Evaluation of DHI Framework |
|  | Personalization | Design and Evaluation of DHI Framework |
|  | Content Completeness | Clinical Information Systems Success Model (CISSM) |
|  | Data quality | Comprehensive evaluation framework for telemedicine implementation |
|  | Information quality (relevancy, usefulness, completeness, etc.) | Hospital Information System Success Framework |
|  | Quality of user documentation | Hospital Information System Success Framework |
|  | Information Quality (measures of IS output) | Human, Organization, Process and Technology-fit (HOPT-FIT) |
|  | Timeliness | Khoja–Durrani–Scott Evaluation Framework |
|  | Accuracy | Khoja–Durrani–Scott Evaluation Framework |
|  | Efficiency/error rates | Khoja–Durrani–Scott Evaluation Framework |
|  | Technical safety (technical reliability) | Model for Assessment of Telemedicine (Mast Manual) |
|  | Clinical safety (patients and staff) | Model for Assessment of Telemedicine (Mast Manual) |
|  | Security | Infoway benefits evaluation Framework |
|  | Security | Evaluation Framework for Fit-For-Purpose Connected Sensor Technologies |
|  | Safety | Design and Evaluation of DHI Framework |
|  | Clinical safety | Health technology assessment framework for digital healthcare services (Digi HTA) |
|  | Security | Digital Health Score Card |
|  | Safety | Development of an Evaluation Framework for Health Information Systems (DIPSA Framework |
|  | Safety | Health Technology Adoption Framework |
|  | System security | Hospital Information System Success Framework |
|  | Clinical Safety | Khoja–Durrani–Scott Evaluation Framework |
|  | Security | Khoja–Durrani–Scott Evaluation Framework |
|  | Security | The layered telemedicine implementation model |
|  | Technical characteristics (eg.) Infrastructure requirements; Interoperability: Integration needs | Model for Assessment of Telemedicine (Mast Manual) |
|  | Interoperability | Health technology assessment framework for digital healthcare services (Digi HTA) |
|  | Interoperability | Comprehensive evaluation framework for telemedicine implementation |
|  | Integration with Legacy system | Hospital Information System Success Framework |
|  | Interoperability and Interconnectivity | Hospital Information System Success Framework |
|  | Using proper standards, coding and nomenclature | Hospital Information System Success Framework |
|  | Interoperability and standardization | Khoja–Durrani–Scott Evaluation Framework |
|  | Interoperability | Digital Health Score Card |
|  | Knowledge needed to use | Nonadoption, abandonment, scale-up, spread, and sustainability Framework (NASSS) |
|  | Availability | Infoway benefits evaluation Framework |
|  | Product information (detailed information about the product e.g. name, technology readiness or intended use) | Health technology assessment framework for digital healthcare services (Digi HTA) |
|  | Accessibility | Health technology assessment framework for digital healthcare services (Digi HTA) |
|  | Accessibility | Clinical Information Systems Success Model (CISSM) |
|  | Access | Health Technology Adoption Framework |
|  | Sustainability | Health Technology Adoption Framework |
|  | Flexibility towards dynamic changes and changes in the organizational context | Hospital Information System Success Framework |
|  | Flexible (can be modified to suit local cultural/social needs) | Khoja–Durrani–Scott Evaluation Framework |
|  | Easily adaptable to different settings (patenting) | Khoja–Durrani–Scott Evaluation Framework |
|  | Appropriate in a variety of conditions | Khoja–Durrani–Scott Evaluation Framework |
|  | Ability to be incorporated into policy | Khoja–Durrani–Scott Evaluation Framework |
|  | Acceptability of e-health | Khoja–Durrani–Scott Evaluation Framework |
| **Objective Value/Effect** | Supply-side value (to developer) | Nonadoption, abandonment, scale-up, spread, and sustainability Framework (NASSS) |
|  | Demand-side value (desirability, efficacy, effectiveness) | Nonadoption, abandonment, scale-up, spread, and sustainability Framework (NASSS) |
|  | Clinical Effectiveness: | Model for Assessment of Telemedicine (Mast Manual) |
|  | Effects on mortality | Model for Assessment of Telemedicine (Mast Manual) |
|  | Effects on morbidity | Model for Assessment of Telemedicine (Mast Manual) |
|  | Physical health | Model for Assessment of Telemedicine (Mast Manual) |
|  | Mental health | Model for Assessment of Telemedicine (Mast Manual) |
|  | Effects on health related quality of life (HRQL) | Model for Assessment of Telemedicine (Mast Manual) |
|  | Behavioural outcomes (e.g. exercise) | Model for Assessment of Telemedicine (Mast Manual) |
|  | Utilization of health services | Model for Assessment of Telemedicine (Mast Manual) |
|  | Quality | Infoway benefits evaluation Framework |
|  | Access | Infoway benefits evaluation Framework |
|  | Productivity | Infoway benefits evaluation Framework |
|  | Patient outcome | Health Information Technology Evaluation Framework (HITREF) |
|  | Patient related knowledge | Health Information Technology Evaluation Framework (HITREF) |
|  | Efficiency | Health Information Technology Evaluation Framework (HITREF) |
|  | Appropriateness of patient care | Health Information Technology Evaluation Framework (HITREF) |
|  | Organizational or social quality | Health Information Technology Evaluation Framework (HITREF) |
|  | Evidence based medicine | The layered telemedicine implementation model |
|  | Utility | Evaluation Framework for Fit-For-Purpose Connected Sensor Technologies |
|  | Important outcomes | RE-AIM (Reach, Effectiveness, Adoption, Implementation, and Maintenance) (expanded to clinical informatics) |
|  | Quality of life | RE-AIM (Reach, Effectiveness, Adoption, Implementation, and Maintenance) (expanded to clinical informatics) |
|  | Long-term effects of a program on outcomes | RE-AIM (Reach, Effectiveness, Adoption, Implementation, and Maintenance) (expanded to clinical informatics) |
|  | Time and Efficiency | Adapted nursing care performance framework |
|  | Nurses’ Practice Environment | Adapted nursing care performance framework |
|  | Nursing Processes | Adapted nursing care performance framework |
|  | Nursing-Sensitive Outcomes | Adapted nursing care performance framework |
|  | Effectiveness | Design and Evaluation of DHI Framework |
|  | Effectiveness | Health technology assessment framework for digital healthcare services (Digi HTA) |
|  | Net Benefits | Clinical Information Systems Success Model (CISSM) |
|  | Quality of care | Comprehensive evaluation framework for telemedicine implementation |
|  | Efficacy (evidence based medicine, clinical outcomes and quality of life) | Health Technology Adoption Framework |
|  | Population health (burden of disease) | Health Technology Adoption Framework |
|  | Standard of care | Health Technology Adoption Framework |
|  | Improved DALYs | Khoja–Durrani–Scott Evaluation Framework |
|  | Improved QALYs | Khoja–Durrani–Scott Evaluation Framework |
|  | Direct benefits to users in routine work | Khoja–Durrani–Scott Evaluation Framework |
|  | Benefits in learning | Khoja–Durrani–Scott Evaluation Framework |
|  | Beneficence/nonmaleficence (client, provider, organization) | Khoja–Durrani–Scott Evaluation Framework |
|  | Improved diagnosis and treatment of disease conditions | Khoja–Durrani–Scott Evaluation Framework |
|  | Improved decision support and clinical | Khoja–Durrani–Scott Evaluation Framework |
|  | care and health management | Khoja–Durrani–Scott Evaluation Framework |
|  | Improved access to care | Khoja–Durrani–Scott Evaluation Framework |
|  | Improved quality of care | Khoja–Durrani–Scott Evaluation Framework |
|  | Functional independence among staff | Khoja–Durrani–Scott Evaluation Framework |
|  | Equity of care | Khoja–Durrani–Scott Evaluation Framework |
|  | Stability of services | Khoja–Durrani–Scott Evaluation Framework |
|  | Effects on the delivery of medical care | Khoja–Durrani–Scott Evaluation Framework |
|  | Health impact leading to | Khoja–Durrani–Scott Evaluation Framework |
|  | change in disease status | Khoja–Durrani–Scott Evaluation Framework |
|  | Social impact due to improved access and quality of services | Khoja–Durrani–Scott Evaluation Framework |
|  | Improvement in quality of life | Khoja–Durrani–Scott Evaluation Framework |
|  | Health impact showing change via indicators | Khoja–Durrani–Scott Evaluation Framework |
|  | Unintended Consequences/Benefits | Health Information Technology Evaluation Framework (HITREF) |
|  | Potential negative effects | RE-AIM (Reach, Effectiveness, Adoption, Implementation, and Maintenance) (expanded to clinical informatics) |
|  | Evidence | Digital Health Score Card |
|  | Added functionality are provided by the HIS, enabling users to provide new or better services | Hospital Information System Success Framework |
|  | Verification, analytical validation, and clinical validation | Evaluation Framework for Fit-For-Purpose Connected Sensor Technologies |
| **Individual** | Satisfaction and acceptance | Model for Assessment of Telemedicine (Mast Manual) |
|  | User Satisfaction | Infoway benefits evaluation Framework |
|  | Attitude (towards technology) | The layered telemedicine implementation model |
|  | User satisfaction | Health Information Technology Evaluation Framework (HITREF) |
|  | Patient satisfaction with EHR | Health Information Technology Evaluation Framework (HITREF) |
|  | Patient satisfaction with care | Health Information Technology Evaluation Framework (HITREF) |
|  | Professional Satisfaction | Adapted nursing care performance framework |
|  | Nurse Satisfaction | Clinical Information Systems Success Model (CISSM) |
|  | Comfort with Patient communication (provider) | Comprehensive evaluation framework for telemedicine implementation |
|  | Comfort with Provider interaction (provider) | Comprehensive evaluation framework for telemedicine implementation |
|  | Stakeholder, user and patient satisfaction | Hospital Information System Success Framework |
|  | Intention to use | Infoway benefits evaluation Framework |
|  | Individuals who are willing to participate | RE-AIM (Reach, Effectiveness, Adoption, Implementation, and Maintenance) (expanded to clinical informatics)) |
|  | Resistance to Change (provider) | Comprehensive evaluation framework for telemedicine implementation |
|  | User acceptance (perceived system ease of use, perceived system usefulness) | Hospital Information System Success Framework |
|  | User acceptance | Khoja–Durrani–Scott Evaluation Framework |
|  | Staff (change of role or identity) | Nonadoption, abandonment, scale-up, spread, and sustainability Framework (NASSS) |
|  | Patient (simple vs complex input - expectation) | Nonadoption, abandonment, scale-up, spread, and sustainability Framework (NASSS) |
|  | Carers (available nature of input - assumptions about them) | Nonadoption, abandonment, scale-up, spread, and sustainability Framework (NASSS) |
|  | User habit (patient) | Comprehensive evaluation framework for telemedicine implementation |
|  | User involvement | Hospital Information System Success Framework |
|  | User engagement and commitment | Hospital Information System Success Framework |
|  | Resistance to changes | Hospital Information System Success Framework |
|  | Motivational activities | Hospital Information System Success Framework |
|  | "Involvement" of end user in requirements elicitation phase, selection of vendor, solution, evaluation, features, etc. | Khoja–Durrani–Scott Evaluation Framework |
|  | Clinical involvement in HIT Selection, Implementation, Training | Health Information Technology Evaluation Framework (HITREF) |
|  | Use/behaviour pattern | Infoway benefits evaluation Framework |
|  | Self-reported use | Infoway benefits evaluation Framework |
|  | Clients use | RE-AIM (Reach, Effectiveness, Adoption, Implementation, and Maintenance) (expanded to clinical informatics)) |
|  | Adherence | Design and Evaluation of DHI Framework |
|  | System Use | Human, Organization, Process and Technology-fit (HOPT-FIT) |
|  | Adoption/adaptation of technology on a wider Scale | Khoja–Durrani–Scott Evaluation Framework |
|  | Wide reach | Khoja–Durrani–Scott Evaluation Framework |
|  | Patients Confidence (in the treatment) | Model for Assessment of Telemedicine (Mast Manual) |
|  | Perceived benefit | Design and Evaluation of DHI Framework |
|  | Perceived enjoyment | Design and Evaluation of DHI Framework |
|  | Perceived Usefulness | Clinical Information Systems Success Model (CISSM) |
|  | Comfort with Work flow | Comprehensive evaluation framework for telemedicine implementation |
|  | Location/ travel time (patient) | Comprehensive evaluation framework for telemedicine implementation |
|  | Patient awareness (patient) | Comprehensive evaluation framework for telemedicine implementation |
|  | Understanding of information | Model for Assessment of Telemedicine (Mast Manual) |
|  | Ability to use the application | Model for Assessment of Telemedicine (Mast Manual) |
|  | Empowerment, self-efficacy | Model for Assessment of Telemedicine (Mast Manual) |
|  | Competency | Infoway benefits evaluation Framework |
|  | Perceived Behavioural Control | Clinical Information Systems Success Model (CISSM) |
|  | CIS Use Dependency |  |
|  | User knowledge and skills | Hospital Information System Success Framework |
|  | Autonomy (client based) | Khoja–Durrani–Scott Evaluation Framework |
|  | Degree of adolescent control (autonomy) |  |
|  | Individual readiness to change | Khoja–Durrani–Scott Evaluation Framework |
|  | Expertise on ICT (provider) | Comprehensive evaluation framework for telemedicine implementation |
|  | ICT skills & knowledge (patient) | Comprehensive evaluation framework for telemedicine implementation |
| **Organisation** | Work needed to implement change | Nonadoption, abandonment, scale-up, spread, and sustainability Framework (NASSS) |
|  | Organisational Strategy | Infoway benefits evaluation Framework |
|  | Diffusion | Health Information Technology Evaluation Framework (HITREF) |
|  | Consistency of delivery as intended and the time of the intervention | RE-AIM (Reach, Effectiveness, Adoption, Implementation, and Maintenance) (expanded to clinical informatics)) |
|  | Extent to which a program or policy becomes institutionalized or part of the routine organizational practices and policies | RE-AIM (Reach, Effectiveness, Adoption, Implementation, and Maintenance) (expanded to clinical informatics) |
|  | Make implementation a transparent process within the organization | Hospital Information System Success Framework |
|  | Consider IT implementation as a change process | Hospital Information System Success Framework |
|  | System Implementation | Human, Organization, Process and Technology-fit (HOPT-FIT) |
|  | Penetration/diffusion of innovation (addressing the digital divide) | Khoja–Durrani–Scott Evaluation Framework |
|  | Modification | Khoja–Durrani–Scott Evaluation Framework |
|  | Improvement | Khoja–Durrani–Scott Evaluation Framework |
|  | Customization | Khoja–Durrani–Scott Evaluation Framework |
|  | Policy changes to facilitate broader adoption, implementation, and innovation in e-health | Khoja–Durrani–Scott Evaluation Framework |
|  | Strategy for e-health implementation | Khoja–Durrani–Scott Evaluation Framework |
|  | Capacity to innovate | Nonadoption, abandonment, scale-up, spread, and sustainability Framework (NASSS) |
|  | Readiness for this technology (change) | Nonadoption, abandonment, scale-up, spread, and sustainability Framework (NASSS) |
|  | Nature of adoption (how easy will it be?) | Nonadoption, abandonment, scale-up, spread, and sustainability Framework (NASSS) |
|  | Scope for adaption over time | Nonadoption, abandonment, scale-up, spread, and sustainability Framework (NASSS) |
|  | Organisational resilience | Nonadoption, abandonment, scale-up, spread, and sustainability Framework (NASSS) |
|  | Structure | Model for Assessment of Telemedicine (Mast Manual)Model for Assessment of Telemedicine (Mast Manual) |
|  | Culture | Model for Assessment of Telemedicine (Mast Manual)Model for Assessment of Telemedicine (Mast Manual) |
|  | Culture | Infoway benefits evaluation Framework |
|  | Hardware (availability in the organisation) | Health Information Technology Evaluation Framework (HITREF) |
|  | Organizational culture | Comprehensive evaluation framework for telemedicine implementation |
|  | ICT infrastructure | Comprehensive evaluation framework for telemedicine implementation |
|  | ICT equipment (patient) | Comprehensive evaluation framework for telemedicine implementation |
|  | Resources (e.g. time for training, set-up, implementation, and management) | Comprehensive evaluation framework for telemedicine implementation |
|  | Knowledge and research | Health Technology Adoption Framework |
|  | Organizational stability | Hospital Information System Success Framework |
|  | Rate of hospital independence and authority | Hospital Information System Success Framework |
|  | Organizational capacity for changes | Hospital Information System Success Framework |
|  | Coping with the impact of change | Hospital Information System Success Framework |
|  | resources (human, financial and physical resources and time) | Hospital Information System Success Framework |
|  | Structure | Human, Organization, Process and Technology-fit (HOPT-FIT) |
|  | Environment | Human, Organization, Process and Technology-fit (HOPT-FIT) |
|  | Organisational readiness to change | Khoja–Durrani–Scott Evaluation Framework |
|  | Scope for innovations | Khoja–Durrani–Scott Evaluation Framework |
|  | Human resource factors (management style, working relationship, communications flow, staff motivation) | Khoja–Durrani–Scott Evaluation Framework |
|  | Organizational Support/ Capacity | Health Information Technology Evaluation Framework (HITREF) |
|  | Settings and intervention agents (people who deliver the program) who are willing to initiate a program | RE-AIM (Reach, Effectiveness, Adoption, Implementation, and Maintenance) (expanded to clinical informatics) |
|  | Social Support (to use CIS) | Clinical Information Systems Success Model (CISSM) |
|  | Service Support | Clinical Information Systems Success Model (CISSM) |
|  | Education & Training & Support | Comprehensive evaluation framework for telemedicine implementation |
|  | Training | Health Technology Adoption Framework |
|  | Sufficient training to make the best out of the daily operation | Hospital Information System Success Framework |
|  | Sufficient training to provide an understanding of its limitations and future potentials | Hospital Information System Success Framework |
|  | Training of all staff, including clinical and management staff | Khoja–Durrani–Scott Evaluation Framework |
|  | Support | The layered telemedicine implementation model |
|  | Training | The layered telemedicine implementation model |
|  | (leadership etc.) | Nonadoption, abandonment, scale-up, spread, and sustainability Framework (NASSS) |
|  | Management | Model for Assessment of Telemedicine (Mast Manual)Model for Assessment of Telemedicine (Mast Manual) |
|  | Leadership | Comprehensive evaluation framework for telemedicine implementation |
|  | Change management | Comprehensive evaluation framework for telemedicine implementation |
|  | Participation in decision-making | Hospital Information System Success Framework |
|  | Support from higher level organizations | Hospital Information System Success Framework |
|  | Managers commitment | Hospital Information System Success Framework |
|  | Formulation and expression of a clear vision for the enterprise | Hospital Information System Success Framework |
|  | showing the HIS as part of it | Hospital Information System Success Framework |
|  | Setting clear goals and instructions | Hospital Information System Success Framework |
|  | Flexible planning | Hospital Information System Success Framework |
|  | Prospective and proactive control | Hospital Information System Success Framework |
|  | Having a strategy | Hospital Information System Success Framework |
|  | Handling the diversity within stakeholder goals | Hospital Information System Success Framework |
|  | Using formal project management methodology | Hospital Information System Success Framework |
|  | Dedicate, availability and prioritize of competitive hospital | Hospital Information System Success Framework |
|  | Identify and mitigate risk (risk management) | Hospital Information System Success Framework |
|  | Understanding socio-technical nature of HIS | Hospital Information System Success Framework |
|  | Business Process Management | Human, Organization, Process and Technology-fit (HOPT-FIT) |
|  | Lean Method | Human, Organization, Process and Technology-fit (HOPT-FIT) |
|  | Prioritizing e-health over other issues | Khoja–Durrani–Scott Evaluation Framework |
|  | Plan for change management | Khoja–Durrani–Scott Evaluation Framework |
|  | Effective change management (preparation and action) | Khoja–Durrani–Scott Evaluation Framework |
|  | Effective change management (maintenance) | Khoja–Durrani–Scott Evaluation Framework |
|  | Policies for change management | Khoja–Durrani–Scott Evaluation Framework |
|  | Limited changes in organizational and national policies to facilitate e-health implementation | Khoja–Durrani–Scott Evaluation Framework |
|  | Healthy public policy and organizational practice | Khoja–Durrani–Scott Evaluation Framework |
|  | Collaboration | Development of an Evaluation Framework for Health Information Systems (DIPSA Framework |
|  | Collaboration and cooperation | Hospital Information System Success Framework |
|  | Internal communication and clear feedback | Hospital Information System Success Framework |
|  | Service quality (the support provided by the information department, the support provided by the maintenance company) | Hospital Information System Success Framework |
|  | Knowledge sharing with other organizations and countries | Khoja–Durrani–Scott Evaluation Framework |
|  | Extend of change needed to routines | Nonadoption, abandonment, scale-up, spread, and sustainability Framework (NASSS) |
|  | Process | Model for Assessment of Telemedicine (Mast Manual)Model for Assessment of Telemedicine (Mast Manual) |
|  | Business Process | Infoway benefits evaluation Framework |
|  | Work flow reengineering | Comprehensive evaluation framework for telemedicine implementation |
|  | Procedures | Development of an Evaluation Framework for Health Information Systems (DIPSA Framework |
|  | Service coordination | Health Technology Adoption Framework |
|  | Work from the workflow | Hospital Information System Success Framework |
|  | Regular evaluations and using their results at different stages of HIS life cycle | Hospital Information System Success Framework |
|  | Clinical Flow/Standard | Human, Organization, Process and Technology-fit (HOPT-FIT) |
|  | Service Quality | Human, Organization, Process and Technology-fit (HOPT-FIT) |
|  | Intramural and extramural work practices | The layered telemedicine implementation model |
| **Societal** | Political/ policy | Nonadoption, abandonment, scale-up, spread, and sustainability Framework (NASSS) |
|  | Rules/policy | Comprehensive evaluation framework for telemedicine implementation |
|  | Governmental authority | Comprehensive evaluation framework for telemedicine implementation |
|  | Political games/conflicts | Hospital Information System Success Framework |
|  | (political) Willingness towards investment on IT systems | Hospital Information System Success Framework |
|  | Legislation and Policy | The layered telemedicine implementation model |
|  | security (patient information) (Legislation) | The layered telemedicine implementation model |
|  | Patient privacy | Health Information Technology Evaluation Framework (HITREF) |
|  | Privacy & security | Design and Evaluation of DHI Framework |
|  | Data security and protection | Health technology assessment framework for digital healthcare services (Digi HTA) |
|  | Information security | Comprehensive evaluation framework for telemedicine implementation |
|  | Regulatory/ legal | Nonadoption, abandonment, scale-up, spread, and sustainability Framework (NASSS) |
|  | Legal issues | Model for Assessment of Telemedicine (Mast Manual) |
|  | Data rights and governance | Evaluation Framework for Fit-For-Purpose Connected Sensor Technologies |
|  | Privacy certification & license | Comprehensive evaluation framework for telemedicine implementation |
|  | Privacy & security rule | Comprehensive evaluation framework for telemedicine implementation |
|  | Interface standards | Comprehensive evaluation framework for telemedicine implementation |
|  | Practice Medical liability | Comprehensive evaluation framework for telemedicine implementation |
|  | Compliance with legal requirements | Hospital Information System Success Framework |
|  | Know what the legal constraints/opportunities | Hospital Information System Success Framework |
|  | Justice and equity | Khoja–Durrani–Scott Evaluation Framework |
|  | Liability | Khoja–Durrani–Scott Evaluation Framework |
|  | Licesure | Khoja–Durrani–Scott Evaluation Framework |
|  | Standardisation | The layered telemedicine implementation model |
|  | Professional | Nonadoption, abandonment, scale-up, spread, and sustainability Framework (NASSS) |
|  | Socio-cultural | Nonadoption, abandonment, scale-up, spread, and sustainability Framework (NASSS) |
|  | Social issues | Model for Assessment of Telemedicine (Mast Manual) |
|  | Society | Comprehensive evaluation framework for telemedicine implementation |
|  | Social norms & values | Comprehensive evaluation framework for telemedicine implementation |
|  | Understand health care as a specific culture | Hospital Information System Success Framework |
|  | Understand the local culture (such as attention to cultural differences between public and private hospitals as well as developing and developed countries) | Hospital Information System Success Framework |
|  | Preparedness and willingness towards cultural change (professional culture) | Hospital Information System Success Framework |
|  | (Cultural) Expectations of users | Hospital Information System Success Framework |
|  | Cultural acceptability | Khoja–Durrani–Scott Evaluation Framework |
|  | Environmental viability | Khoja–Durrani–Scott Evaluation Framework |
|  | Gender issue/gender divide | Khoja–Durrani–Scott Evaluation Framework |
|  | Sensitive to sociocultural issues | Khoja–Durrani–Scott Evaluation Framework |
|  | Societal readiness to technology change | Khoja–Durrani–Scott Evaluation Framework |
| **Ethics** | Ethical issues | Model for Assessment of Telemedicine (Mast Manual) |
|  | Ethics | Design and Evaluation of DHI Framework |
|  | Compliance with existing ethical rules in affairs management | Hospital Information System Success Framework |
|  | Privacy and confidentiality |  |
|  | Moral consideration | Khoja–Durrani–Scott Evaluation Framework |
|  | Autonomy (client based) |  |
|  | Scalability | Khoja–Durrani–Scott Evaluation Framework |
| **Economics** | Supply-side value (business case) | Nonadoption, abandonment, scale-up, spread, and sustainability Framework (NASSS) |
|  | Business case (institutional level) | Model for Assessment of Telemedicine (Mast Manual) |
|  | Company information | Health technology assessment framework for digital healthcare services (Digi HTA) |
|  | Return on investment (material or immaterial) | Hospital Information System Success Framework |
|  | Budget (Hospital) | Comprehensive evaluation framework for telemedicine implementation |
|  | Reimbursement | Comprehensive evaluation framework for telemedicine implementation |
|  | 3rd party payer | Comprehensive evaluation framework for telemedicine implementation |
|  | Sufficient funding | Hospital Information System Success Framework |
|  | Reimbursement | Khoja–Durrani–Scott Evaluation Framework |
|  | Funding support for research | Khoja–Durrani–Scott Evaluation Framework |
|  | Funding decision (organisation) | Nonadoption, abandonment, scale-up, spread, and sustainability Framework (NASSS) |
|  | Costs of information processing | Health Information Technology Evaluation Framework (HITREF) |
|  | Cost of the intervention | RE-AIM (Reach, Effectiveness, Adoption, Implementation, and Maintenance) (expanded to clinical informatics) |
|  | Cost | Health technology assessment framework for digital healthcare services (Digi HTA) |
|  | Productivity (net costs/efficiency) | Infoway benefits evaluation Framework |
|  | Medical costs (out of pocket) | Comprehensive evaluation framework for telemedicine implementation |
|  | Purchasing price | Digital Health Score Card |
|  | Anticipated costs | Digital Health Score Card |
|  | Cost (resources, infrastructure) | Health Technology Adoption Framework |
|  | Development cost, availability, affordability | Khoja–Durrani–Scott Evaluation Framework |
|  | Cost | Khoja–Durrani–Scott Evaluation Framework |
|  | Affordability | Khoja–Durrani–Scott Evaluation Framework |
|  | Cost | The layered telemedicine implementation model |
|  | Demand-side value (cost-effectiveness) | Nonadoption, abandonment, scale-up, spread, and sustainability Framework (NASSS) |
|  | Economic evaluation (societal perspective) | Model for Assessment of Telemedicine (Mast Manual) |
|  | Sensitivity analysis (Risk analysis) | Model for Assessment of Telemedicine (Mast Manual) |
|  | Relevant alternatives | Model for Assessment of Telemedicine (Mast Manual) |
|  | Costs of patient care | Health Information Technology Evaluation Framework (HITREF) |
|  | Economic feasibility | Evaluation Framework for Fit-For-Purpose Connected Sensor Technologies |
|  | Economic outcomes | RE-AIM (Reach, Effectiveness, Adoption, Implementation, and Maintenance) (expanded to clinical informatics) |
|  | Cost effectiveness | Comprehensive evaluation framework for telemedicine implementation |
|  | Economic analysis (cost-effectiveness, cost-benefit) | Health Technology Adoption Framework |
|  | Justification of increase of costs | Hospital Information System Success Framework |
|  | Cost benefit | Khoja–Durrani–Scott Evaluation Framework |
|  | Cost minimization | Khoja–Durrani–Scott Evaluation Framework |
|  | Cost-utility | Khoja–Durrani–Scott Evaluation Framework |
|  | Cost-benefit | Khoja–Durrani–Scott Evaluation Framework |
|  | (Financial) Provider and structure/cost effectiveness | The layered telemedicine implementation model |
| **Strategic** | Strategic fit | Health Technology Adoption Framework |
|  | National, regional, organizational (Strategy) | Hospital Information System Success Framework |
|  | Accepted also at lower levels | Hospital Information System Success Framework |
|  | Alignment between system strategies and hospital strategies | Hospital Information System Success Framework |
|  | Reliable external partners | Hospital Information System Success Framework |
|  | Strategy for e-health implementation | Khoja–Durrani–Scott Evaluation Framework |
|  | Problem handling | Khoja–Durrani–Scott Evaluation Framework |
|  | Trust | Khoja–Durrani–Scott Evaluation Framework |
|  | Cross-border (transferability) | Model for Assessment of Telemedicine (Mast Manual) |
|  | Scalability (of results) | Model for Assessment of Telemedicine (Mast Manual) |
|  | Generalizability (of results) | Model for Assessment of Telemedicine (Mast Manual) |
|  | Barriers or Facilitators to Adoption | Health Information Technology Evaluation Framework (HITREF) |
|  | Barriers and facilitators | Khoja–Durrani–Scott Evaluation Framework |
