# Supplementary material for: Evaluation frameworks for digital nursing technologies: analysis, assessment, and guidance. An overview of the literature
Source: BMC Nurs. 2021 Aug 17;20:146. doi: 10.1186/s12912-021-00654-8 (PMC8369663; doi:10.1186/s12912-021-00654-8)
Supplement: Supplementary file 3 — Additional file 3. Detailed strength and weakness analysis. [file 12912_2021_654_MOESM3_ESM.docx]

| Additional file 3 - detailed strength and weakness analysis | | | |  | |
| --- | --- | --- | --- | --- | --- |
|  | | | | **Assessment** | |
|  |  | **Framework** |  | **Strengths** | **Weaknesses** |
|  |  | Infoway benefits evaluation Framework [19] |  | - Clear description of the purpose and addressed question (to evaluate benefits for investments) - Generalistic Health (business) settings described as a setting - Applicable for different health information technologies - Very well illustrated presentation of the model - Visualization of connections and relationships within the framework is given - Clear definition of terms with examples - Full transparency of the development process - Transferable (allows comparison of evaluation findings) | - No clear focus on a specific technology type - No specific application settings described - No application strategy for the framework - No guidance on interpretation of results - No discussion of weaknesses and limitations |
|  |  | Health Information Technology Evaluation Framework (HITREF)[20] |  | - Clear description of the purpose and the addressed question - Clear description of specific technology (health information technologies (EHR)) - Universal approach by referring to different possible settings (hospital, ambulatory, community-based, public health) - Well-illustrated presentation of the model - Visualization of connections and relationships within the framework is given - Clear definition of key concepts and terms - Exemplary explanation for the application - Full transparency of the development process - Transferable to different settings | - No specific application settings described - No guidance on interpretation of results - Insufficient discussion of weaknesses and limitations |
|  |  | Hospital Information System Success Framework [7] |  | - Clear description of the purpose and the addressed question - Clear description of the setting (hospital) - Clear description of the technology (hospital information systems) - Description of key concepts by presenting information on related sub factors and evaluation methods - Presentation of recommended methods and example studies for individual evaluation factors - Full transparency of the development process - Limitations/weaknesses are sufficiently described - Transferability in the hospital setting is given | - No visualization of the framework (table only) - No visualization of connections or relationships within the framework - No guidance on interpretation of results |
|  |  | Development of an Evaluation Framework for Health Information Systems (DIPSA Framework) [21] |  | - Clear description of the purpose and the addressed question - Clear description of the setting (hospital) - Clear description of the technology (integrated Health Information Systems) - Description and definition of key concepts and terms with related questions (questions are not easily accessible) - Application strategy exists - Description of the development process is given - Transferable to other hospital settings | - No visualization of the framework (table only) - No visualization of connections or relationships within the framework - Application of the framework only possible if the questions are available - No concrete instruction on how the results can be interpreted - Insufficient description of the limitations |
|  |  | Human, Organization, Process and Technology-fit (HOPT-FIT) [22] |  | - Clear description of the purpose and addressed question - Applicable for different types of health organisations (setting) - Clear description of the technology (health information systems) - Well-illustrated presentation of the model - Visualization of connections and relationships within the framework is given | - Setting unspecified (health organisation) - Insufficient explanation of the individual terms - No concrete application strategy and instructions for use. The framework is still to be tested in clinical settings - No instruction on how the results can be interpreted - Methodology of the development process not sufficiently described - Insufficient discussion of weaknesses and limitations - Unclear transferability due to weaknesses in the development methodology |
|  |  | Clinical Information Systems Success Model (CISSM) [4] |  | - Clear description of the purpose and the addressed question - Clear description of the setting (hospital) - Clear description of the technology (clinical information systems) - Very well illustrated presentation of the model - Visualization of connections and (statistically validated) relationships within the framework is given - Clear definition of key concepts and terms - Procedure, instruments, and evaluation matrix are given. A validation study has been carried out - Examples are provided to aid interpretation - Full transparency of the development process - Transferable to other hospital settings | - Discussion of weaknesses and limitations very short - Limited transferability of the framework to other settings |
|  |  | Adapted nursing care performance framework [23] |  | - Clear description of the purpose and the addressed question - Clear description of technology (information and communication technologies for nurses) - Broad (healthcare) setting related to nursing care - Well-illustrated presentation of the model - Visualization of connections and relationships within the framework is given - Detailed definition and explanation of terms and concepts based on the studies examined - Description of the analysis process is given - Discussion of the weaknesses, biases, and limitations - Transferability to different nursing contexts given | - No clear focus on a specific on a setting - Framework as an overview with no application strategy - Indirect explanation how results can be interpreted (by referring to analysed studies) - Development process: No independent framework - rather the examination of the transferability of indicators of a framework to ICTs in the field of nursing |
|  |  | Model for Assessment of Telemedicine (MAST Manual) [24] |  | - Clear description of the purpose and the addressed question - Universal setting approach (all telemedicine settings) - Clear description on specific technology (telemedicine technologies -application to other contexts still conceivable) - Clear definition of included domains (e.g. safety) with examples - Concrete application strategy and instructions for use is given including possible methods - High transparency of the development process - Limitations/weaknesses are sufficiently described - Transferability of the framework is described | - Setting undefined (telemedicine) - Moderate clarity of illustration - No visualization of connections or relationships within the framework - No guidance on interpretation of results |
|  |  | Comprehensive evaluation framework for telemedicine implementation  [18] |  | - Clear description of the purpose and the addressed question - Universal setting approach (all telemedicine settings) - Clear description of the technology (telemedicine systems) - Vivid illustration | - Setting undefined (telemedicine) - Lack of clarity of connections or relationships within the framework - Insufficient definition of terms in the framework - No concrete application strategy and instructions for use - No instruction on how the results can be interpreted - Methodology of the development process not sufficiently described - Insufficient description of the limitations - Unclear transferability |
|  |  | The layered telemedicine implementation model [17] |  | - Clear description of the purpose and the addressed question - Universal setting approach (all telemedicine settings) - Clear description of the technology (telemedicine interventions) - Well-illustrated presentation of the model - Visualization of connections and relationships within the framework is given - Clear definition of key concepts and terms - Instruction to choose a layered approach along the development life cycle. Different determinants should gain focus during the maturity of the telemedicine implementation - Explanation of the development process is given - Limitations/weaknesses are sufficiently described | - Setting undefined (telemedicine) - No concrete application strategy and instructions for use - only example studies and the advice to involve multiple stakeholders in the analysis. - No instruction on how the results can be interpreted - Unclear transferability (study provides only an overview of the determinants described in the literature) |
|  |  | Evaluation Framework for Fit-For-Purpose Connected Sensor Technologies [15] |  | - Clear description of the purpose, clear description of the addressed question (risk evaluation) - Applicable for clinical or research settings - Clear description of specific technology area (connected sensors) - Clear illustration - Clear definition of key concepts and terms - Exemplary explanation for the application - Sample threshold criteria for the interpretation of the results - Sufficient explanation of the development process - Transferable to different settings | - No specific application settings described - No visualization of connections or relationships within the framework - No discussion of weaknesses and limitations |
|  |  | Design and Evaluation of DHI Framework [11] |  | - Clear description of the purpose and wide variety of questions to be addressed to - Applicable for different care settings - Applicable for different digital health interventions (universal) - Clear definition of key concepts and terms - Description of the application based on different digital health intervention phases - Full transparency of the development process - Limitations/weaknesses are sufficiently described - Transferable to different settings and technologies | - No clear focus on a specific a technology area - No clear focus on a specific setting - No visualization of the framework (table only) - No visualization of connections or relationships within the framework - No instruction on how the results can be interpreted |
|  |  | Health technology assessment framework for digital healthcare services (Digi HTA) [6] |  | - Clear description of the purpose and the addressed question - Applicable for different care settings - Applicable for different digital healthcare services (mHealth, AI, and robotics) - Evaluation categories (key concepts) are specified by means of questions - Application procedure is described - Grid for the interpretation of the results is presented (guidance on decision) - Full transparency of the development process - Transferable to different settings | - Unspecified setting - No visualization of the framework (table only) - No visualization of connections or relationships within the framework - Insufficient description of the limitations |
|  |  | Digital Health Score Card [10] |  | - Clear description of the purpose and the addressed question - Applicable for different care settings - Applicable for different digital health technologies - Very well illustrated presentation of the model - Visualization of connections within the framework is given - Clear definition of key concepts and terms | - No clear focus on a specific setting - No clear focus on a specific technology type - No concrete application strategy and instructions for use. The framework is still in the early stages of development and iteration is ongoing - No instruction on how the results can be interpreted - Development process not sufficiently described - Insufficient description of the limitations - Unclear transferability |
|  |  | Khoja–Durrani–Scott Framework for e-Health Evaluation [16] |  | - Clear description of the purpose and the addressed question - Universal setting approach (e-health) - Broad spectrum of possible technologies to be evaluated (e-health) - Table illustrates the relationships in the framework based on the e-health life cycle - Important terms and concepts are defined - but not every term is sufficiently defined in the framework - Sufficient explanation of the development process - Transferable to different settings and technologies | - Setting undefined (e-health) - No clear focus on technology area (e-health) - No visualization of the framework (table only) - No concrete application strategy and instructions for use (Specific evaluation tools were developed, but were not publicly available at the time of the review) - No instruction on how the results can be interpreted - No discussion of weaknesses and limitations |
|  |  | RE-AIM (Reach, Effectiveness, Adoption, Implementation, and Maintenance) (expanded to clinical informatics) [25] |  | - Clear description of the purpose and research question (how research can be translated into practice) - Universal approach (applicable for “clinical informatics”) - Clear description of the framework concepts and terms - Examples for the application given - Example advice for interpretation – (not very concrete) - Development: Framework based on the original article and validated by clinical information case studies - Meta Framework (very transferable) | - No clear focus on a specific technology area - No clear focus on a specific on a setting (“clinical informatics”) - Illustration is a table - No visualization of connections or relationships within the framework - Insufficient discussion of weaknesses and limitations |
|  |  | Health Technology Adoption Framework [8] |  | - Clear description of the purpose and the addressed question - Clear description of the setting (surgical context) - Clear description of the technology area is given (health technologies in the surgical context) - Vivid illustration with evaluation tool - Definitions of the domains, criteria and sub criteria provided (related questions provided) - Concrete application strategy and instructions for use is given by presenting an evaluation tool - Instruction on how the results can be interpreted is included in the evaluation tool - Detailed description of the development process is given - Limitations/weaknesses are sufficiently described - Transferable to other surgical settings | - No visualization of connections or relationships within the framework - Transferability limited (limited to surgical context) |
|  |  | Nonadoption, abandonment, scale-up, spread, and sustainability Framework  (NASSS Framework) [9] |  | - Clear description of the purpose and the addressed question - Applicable for different care settings - Broad spectrum of possible technologies to be evaluated (Health and care technologies) - Well-illustrated presentation of the model - Visualization of connections and relationships within the framework is given - Clear definition of terms and associated question within the framework - Detailed examples of usage possibilities - Classification system to interpret the results (simple, complicated, complex) - Sound science-based framework development - Limitations are sufficiently described - High transferability to a wide variety of settings through generalistic structure | - No clear focus on a specific technology type - No clear focus on a specific setting - The individual application strategy of the framework must always be reflected, as it is not a directly applicable or a formulaic instrument |
